# Supplementary material for: How myosin VI traps its off-state, is activated and dimerizes
Source: Nat Commun. 2023 Oct 23;14:6732. doi: 10.1038/s41467-023-42376-2 (PMC10593786; doi:10.1038/s41467-023-42376-2)
Supplement: Supplementary file 1 — Supplementary Information [file 41467_2023_42376_MOESM1_ESM.pdf]

Supplementary information

How Myosin VI Traps its Off-State, is Activated and Dimerizes

Louise Canon[1], Carlos Kikuti[1], Vicente J. Planelles-Herrero[1], Tianming Lin[2], Franck Mayeux[1], Helena Sirkia[1], Young il Lee[2], Leila Heidsieck[1], Léonid Velikovsky[1], Amandine David[1], Xiaoyan Liu[2], Dihia Moussaoui[1], Emma Forest[1,3], Peter Höök[2], Karl J. Petersen[1], Tomos E. Morgan[4], Aurélie Di Cicco[5], Julia Sires-Campos[6], Emmanuel Derivery[4], Daniel Lévy[5], Cédric Delevoye[6], H. Lee Sweeney[2,*], Anne Houdusse[1,*]

SUMMARY

**Supplementary Figures**	**1-19**	**pages 2-25**
**Supplementary Text for crosslinking mass spectrometry**		**page 26**
**Supplementary Tables**	**1-4**	**pages 27-33**
**Supplementary References**		**pages 34**

Supplementary Figures

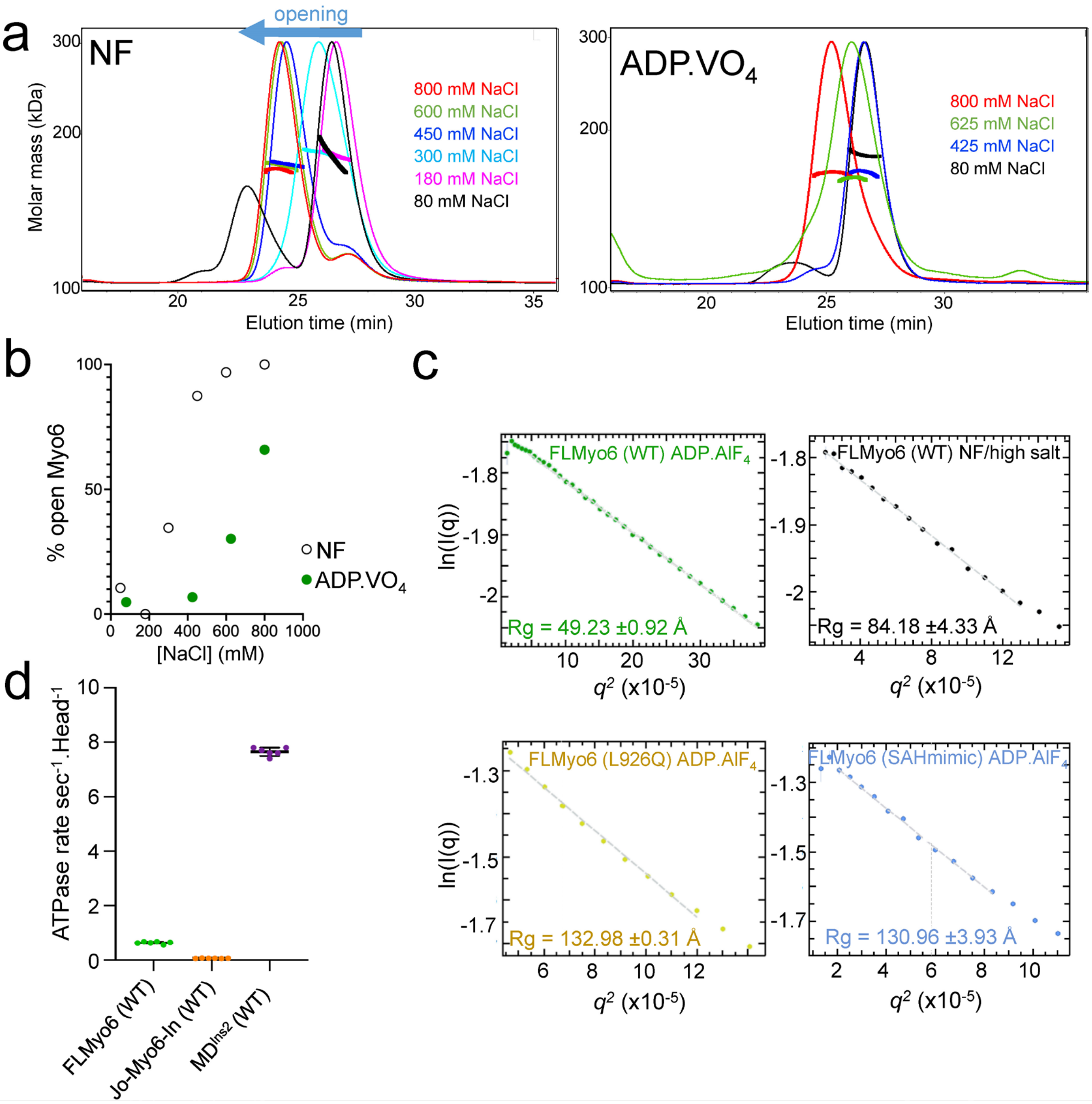

Supplementary Figure 1 – ATP is required for the compact, back-folded conformation of Myo6.

(a) Normalized SEC-MALS profiles of FLMyo6 in different elution buffers. Thin lines: normalized static light scattering; Thick lines: molecular mass as determined for each 0.5 second experimental point by Astra (Wyatt technology). ADP.VO$_4$ buffer: 20 mM Hepes; 2 mM MgCl$_2$; 1 mM NaADP; 1 mM NaVO$_4$; 0.1 mM EGTA; 0.5 mM TCEP, pH 7.5 + indicated NaCl concentration; NF buffer: 20 mM Hepes; 2 mM MgCl$_2$; 0.1 mM EGTA; 0.5 mM TCEP, pH 7.5 + indicated NaCl concentration.

(b) Percentage of open FLMyo6 as a function of [NaCl] in NF and ADP.VO$_4$. Elution times from the two series in Sup Fig. 1A were normalized as follows: (1) the longest retention time of the entire series (180 mM NaCl in NF) was defined as 0% open FLMyo6, and the shortest retention time (800 mM NaCl in NF), as 100% open FLMyo6; (2) Ve/Vo was calculated for each data point; (3) the shifts of Ve/Vo from the minimum were employed as powers of 10 for coherence with the expected relationship between retention time and Rh in SEC; (4) Percentage of closed FLMyo6 was calculated by dividing each shift by the complete range, also as a power of 10; (5) Percentage of open FLMyo6 was calculated by subtracting %closed from one. The entire calculation can be summarized by the following equation:

$$\%\text{open} = 100 * (1 - \left(\frac{10^{\frac{Ve}{Vo}} - 10^{\frac{Ve}{Vo}\text{min}}}{10^{\frac{Ve}{Vo}\text{max} - \frac{Ve}{Vo}\text{min}}} \right))$$

(c) Guinier plot of FLMyo6 WT, SAHmimic and L926Q in ADP.AlF_4 or in NF/high salt. Rg values were extracted from the linear fit (dashed line) using primusqt (ATSAS suite[1]). Rg value ± standard deviation is depicted for each condition. See scattering profiles in Sup Fig. 2.

(d) Actin-activated ATPase rate of 150 nM FLMyo6 (WT), Jo-Myo6-In, and MD^{Ins2} (n=6 from 2-3 preps with 2-3 independent assays per prep) at 40 µM F-actin. Error bars = standard deviation.

(a-d) Source data are provided as a Source Data file.

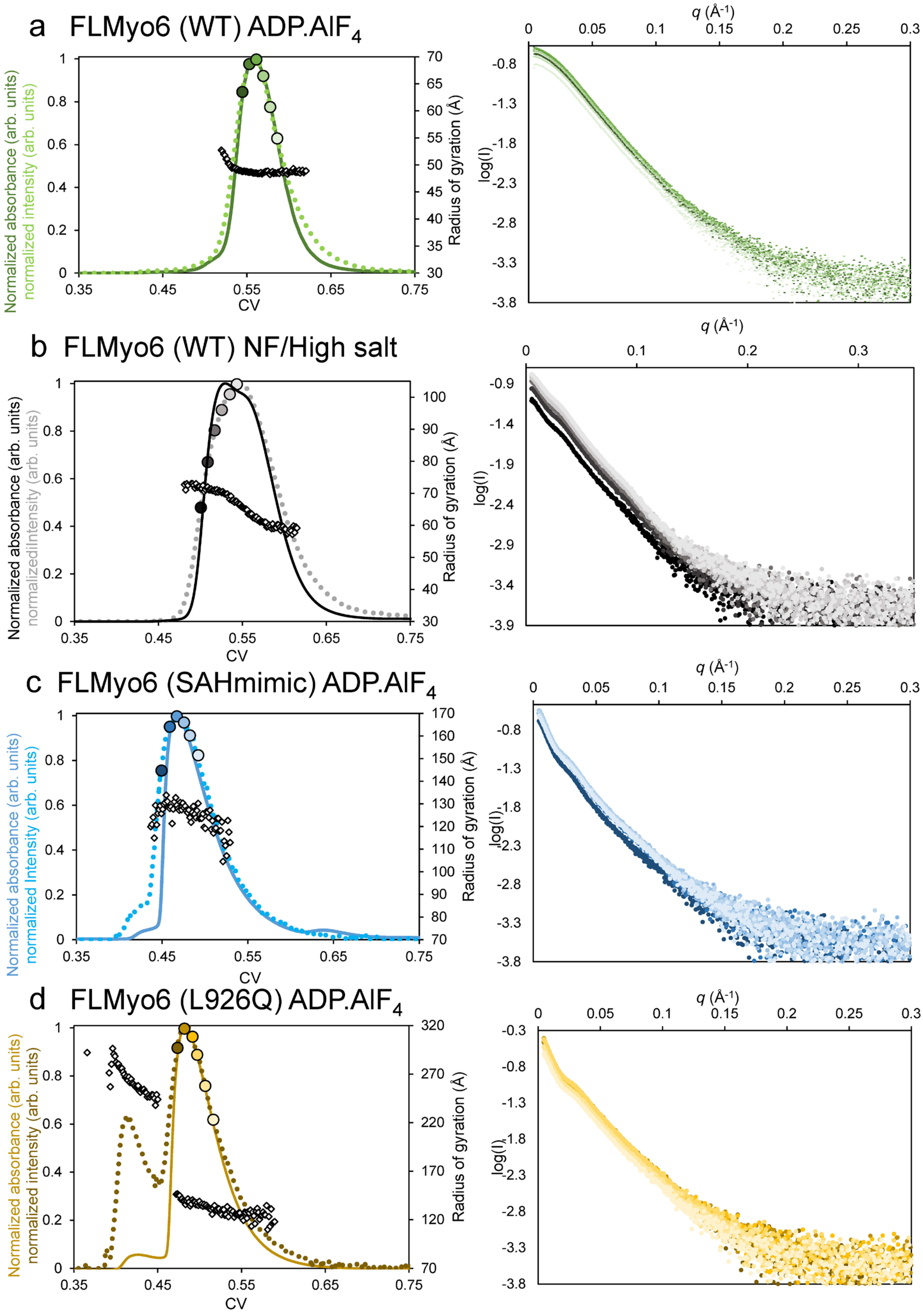

Supplementary Figure 2 – FLMyo6 scattering data

SEC-SAXS data indicate FLMyo6 rearrangements depending on the presence of salt, nucleotide, or mutations. (Left) SEC elution profile of FLMyo6, the normalized absorbance (at 280 nm) is plotted as solid line, the normalized scattering intensity per frame is plotted as dotted line and the radius

of gyration (R_g) per frame are plotted as black squares. Note that in SEC-MALS all the constructs tested here elute as monomers (Sup Fig. 4A) (Right) Scattering profiles (log(I) vs q) for several frames along the peak (highlighted as colored dots).

(a) SEC-SAXS data for FLMyo6 (wild-type) in ADP.AlF$_4$ buffer (see Methods). The peak is symmetric and the R_g is very regular along the peak.

(b) SEC-SAXS data for FLMyo6 (wild-type) in NF-high salt buffer (see Methods). FLMyo6 elutes earlier in NF/high salt condition than in FLMyo6 (wild-type) in ADP.AlF$_4$ condition (as also seen in SEC-MALS, Sup Fig. 1A). Within the first part of the peak, the R_g is pretty regular along the peak then drops by ~20 Å between 0.55 and 0.6 column volume (CV). This possibly indicates the presence of some FLMyo6 that would be only partially opened or possibly closed even in NF/high salt conditions. To characterize the open FLMyo6, only the frames corresponding to the first part of the peak have been considered for R_g calculation and Kratky plot generation.

(c) SEC-SAXS data for FLMyo6 (SAHmimic) in ADP.AlF$_4$ buffer (see Methods). The peak elutes earlier for the mutant than for the wild-type Myo6 (as also seen in SEC-MALS, Sup Fig. 1A). The R_g is stable at the center of the peak and less precise at the edges. Thus, to characterize the open FLMyo6, only the frames corresponding to the center of the peak have been considered for R_g calculation and Kratky plot generation.

(d) SEC-SAXS data for FLMyo6 (L926Q) in ADP.AlF$_4$ buffer (see Methods). The peaks elute earlier for the mutant than for the wild-type Myo6 (as also seen in SEC-MALS, Sup Fig. 1A). Along the main peak, the R_g is around ~130 Å as for the SAHmimic mutant. But here we note the presence of an additional peak between 0.4 and 0.45 CV with a high scattering intensity and a very high and unstable R_g. The peak very likely corresponds to some aggregation. It thus was not considered for R_g calculation and Kratky plot generation. Please note that the aggregation peak has a very low absorbance at 280 nm indicating that only a minor part of the protein is aggregated.

(a-d) Source data are provided as a Source Data file.

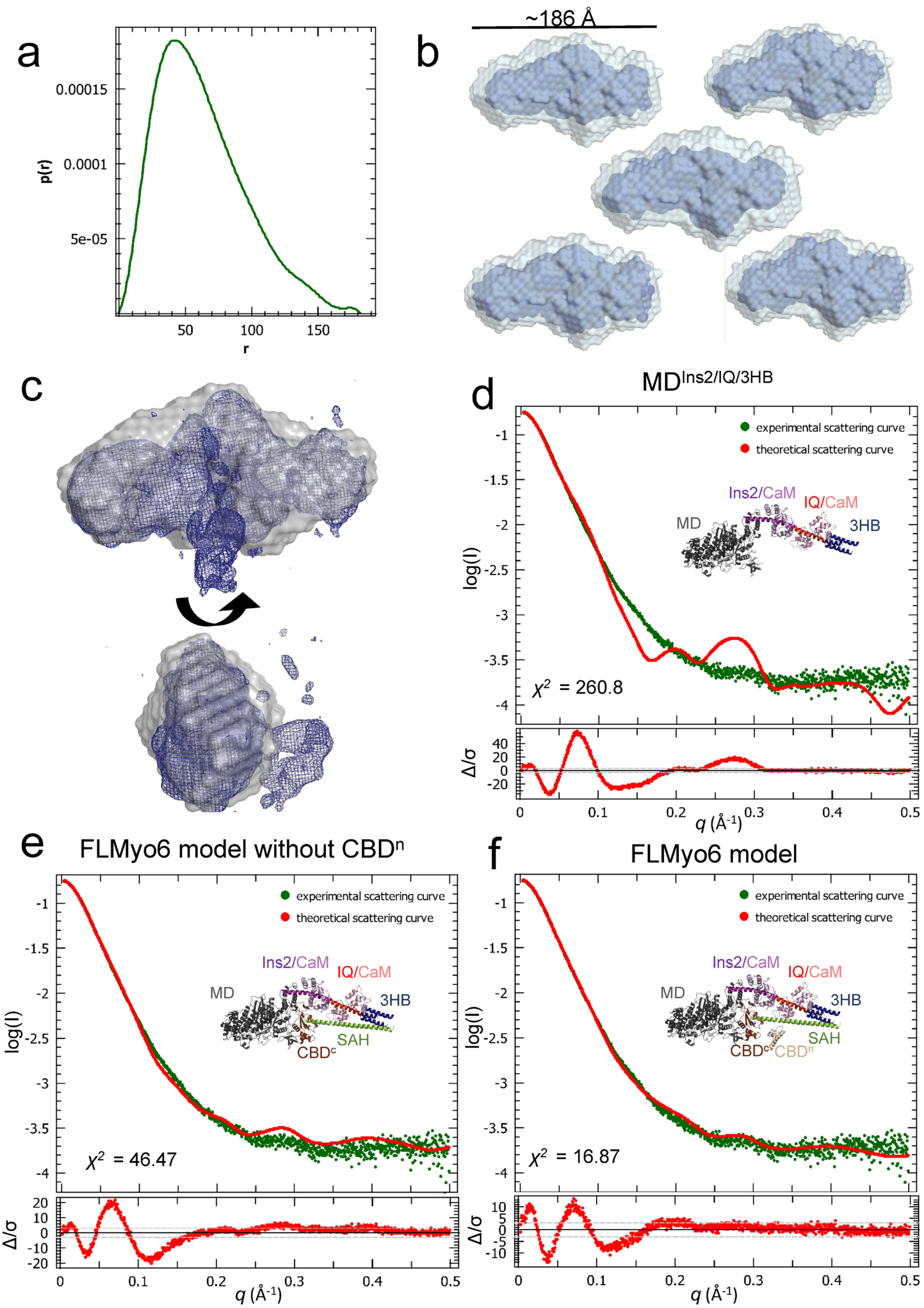

Supplementary Figure 3 - FLMyo6 scattering data interpretation

(a) Pair distance distribution function (P(r)) of FLMyo6 (wild-type) in ADP.AlF_4 buffer (Dmax = 182 Å) used for ab initio reconstructions.

(b) From this P(r) function, 20 ab initio models were generated using GASBOR[2]. A cluster of five of these models is shown in blue as examples. The final envelope is presented in Fig. 1C (obtained by

averaging all the GASBOR generated models using DAMAVER[3]) and is also plotted in grey for comparison. All the models exhibit a similar shape; (out of the 20 models generated, DAMCLUST[4] only identified one cluster of models). Moreover, their analysis[5,6] shows that all of them exhibit a D_{max} range of 177-185 Å and a R_g range of 48.44-49.56 Å, similar to the ones obtained from the scattering curve and P(r) function of FLMyo6 (wild type) in AlF_4 (182 Å and 49.23 Å respectively).
(c) SAXS envelope of FLMyo6 (grey) was manually docked into the negative staining 3D-reconstruction of the Jo-Myo6-In (deep blue). This comparison shows similar size and shape for both although the negative staining 3D-reconstruction displays more details and an additional blob corresponding to the Jo/In domain.
(d-f) Next we compared the FLMyo6 off-state atomic model built based on the stained EM data from the Jo-Myo6-In construct to these SAXS measurements. Using CRYSOL[7], the theoretical scattering curves of the Myo6 off-state atomic models (red) were generated and compared to the experimental SAXS scattering curve of FLMyo6 (wild-type) in ADP.AlF_4 buffer (green). Residuals are plotted in the bottom part of the figure.
(d) The $MD^{Ins2/IQ/3HB}$ model (as seen in Fig. 1C) fits the experimental data very poorly certainly due to the absence of the Myo6 tail in this model
(e-f) Addition of the SAH, CBD^c and CBD^n domains improves the fitting to the experimental scattering data.
(a, d-f) Source data are provided as a Source Data file.

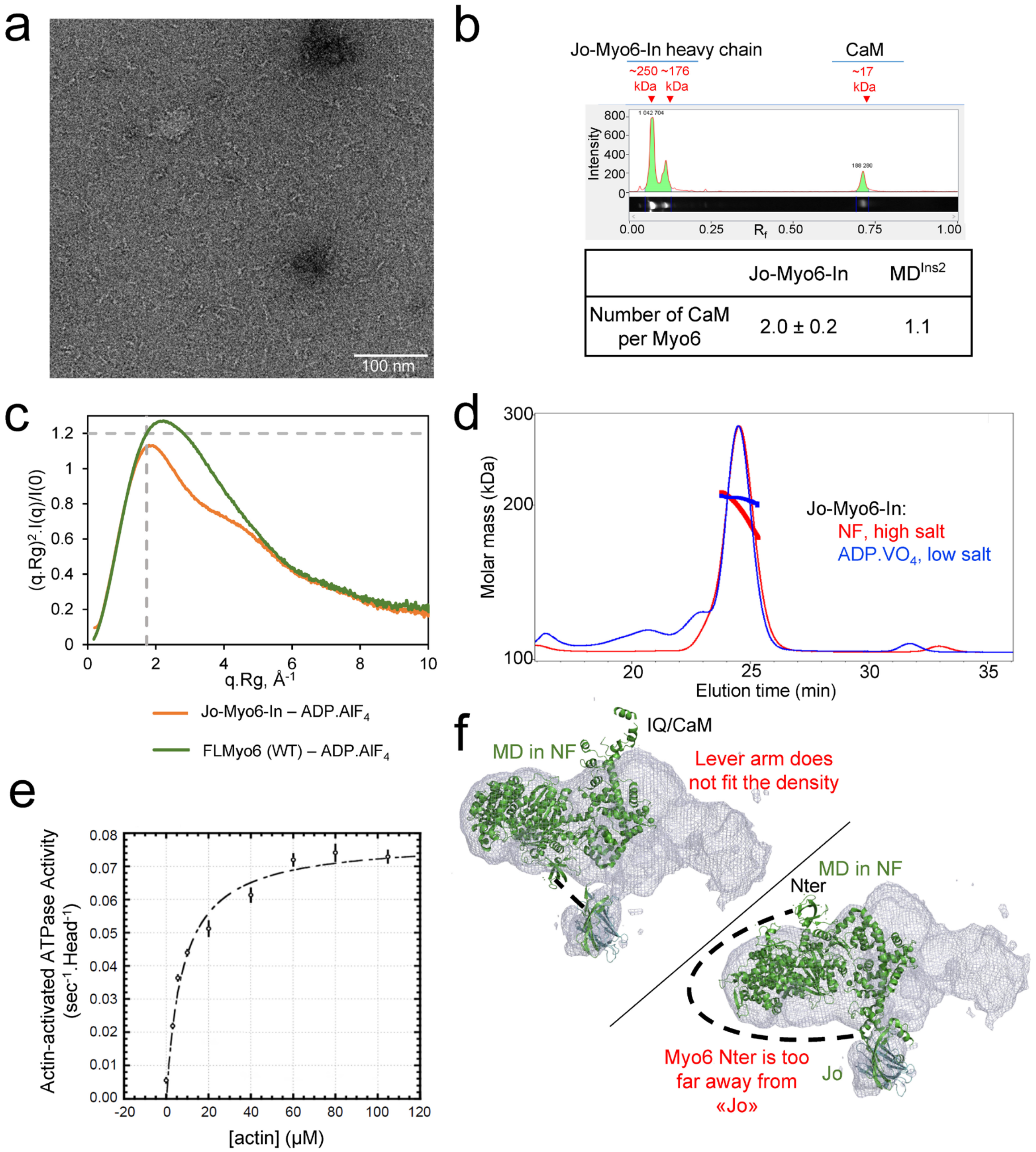

Supplementary Figure 4 – The Jo-In fusion stabilizes the off-state with no effect on its conformation. **(a)** Negative staining image of FLMyo6 without the Jo-In fusion. Purified FLMyo6 was diluted to 50 µg/ml in buffer containing MgADP.VO4 and used for preparing negative staining grids with 2 % uranyl acetate following standard procedure. Images were collected with a Tecnai G2 at 200 kV. 22 grids done with 3 different protein batches.

(b) SDS-PAGE profile of Jo-Myo6-In stained with SYPRO[8]. The estimated molecular weight based on SDS-PAGE is given in red. The CaM/FLMyo6 molar ratio was determined from the intensities of the corresponding bands (Table: mean ± standard deviation from 4 replicates). Intensity of Myo6 and CaM bands were estimated on ImageLab software (Bio-Rad). Ratio CaM/FLMyo6 was estimated by calculating (CaM band intensity)/(Myo6 band intensity) and normalized by theoretical molecular weight to get a molar ratio. MD^{Ins2} was used as a control as it binds 1 CaM per Myo6[9]. Note that, on SDS-PAGE, purified Jo-Myo6-In-Flag appears as two distinct bands, one at its expected molecular weight 176 kDa, and another band around 250 kDa. On SEC, the faster band was found to elute slightly earlier, in a broader peak merged to the one corresponding to the slower band. LC-MS concluded that both bands correspond to Jo-Myo6-In with 68.5% sequence coverage. Exactly the same peptides were

found in both bands, and the Jo and In peptides containing the expected covalent Lys-Asn bond could not be found. Therefore, we assume that the slower band corresponds to the protein whose covalent Lys-Asn bond is correctly formed (=”locked”), whereas the faster band is composed by folded, but not locked, protein (=”closed”).

(c) Dimensionless Kratky plot representation. Dotted lines ($(qRg)^2 I(q)/I(0) = 1.104$ and $qRg = \sqrt{3}$) intersection highlight the theoretical maximum of a globular protein. In the presence of ADP.AlF_4, FLMyo6 (green) and Jo-Myo6-In (orange) spectra result in a bell-shape curve (with a shoulder in the case of Jo-In) with a maximum close to ($\sqrt{3}$:1.104), suggesting that both proteins are rather globular and well folded. The shoulder in the Jo-Myo6-In profile is typical of multi-domain proteins consistent with the addition of the Jo-In tag. See scattering profiles in Sup Fig. 5A.

(d) Normalized SEC-MALS profiles of Jo-Myo6-In in conditions that favor opening (red, NF) or closure (blue, ADPVO_4). NF buffer: 20 mM Hepes; 300 mM NaCl; 2 mM $CaCl_2$; 0.1 mM EDTA; 0.5 mM TCEP, pH 7.5. ADP.VO_4 buffer: 20 mM Heps; 80 mM NaCl; 2 mM $MgCl_2$; 1 mM NaADP; 1 mM NaVO_4; 0.1 mM EGTA; 0.5 mM TCEP, pH 7.5.

(e) Actin-activated ATPase rate of Jo-Myo6-In as a function of actin concentration. Mean values ± standard deviation are plotted. ATPase measurements were performed as outlined in Methods. The V_{max} extrapolates to ~0.08 sec^{-1} and the K_{ATPase} (apparent actin affinity) is ~7 µM actin (ΔG° ~-7 kcal/mol).

(f) Docking of nucleotide-free Myo6 $MD^{Ins2/IQ}$ (PDB: 2BKI[9] [https://doi.org/10.2210/pdb2BKI/pdb]) and Jo-In (PDB: 5MKC[10] [https://doi.org/10.2210/pdb5MKC/pdb]) in the negative staining map. The structure of the Motor domain devoid of nucleotide doesn’t fit the map: **(left)** if the connection between the N-terminus of Myo6 and the C-terminus of Jo is respected, the Lever arm does not fit in the envelope; **(right)** if the Lever arm is positioned inside the 3D reconstruction, the distance between the Myo6 N-terminus and the fusion subdomain Jo is prohibitive.

(b-e) Source data are provided as a Source Data file.

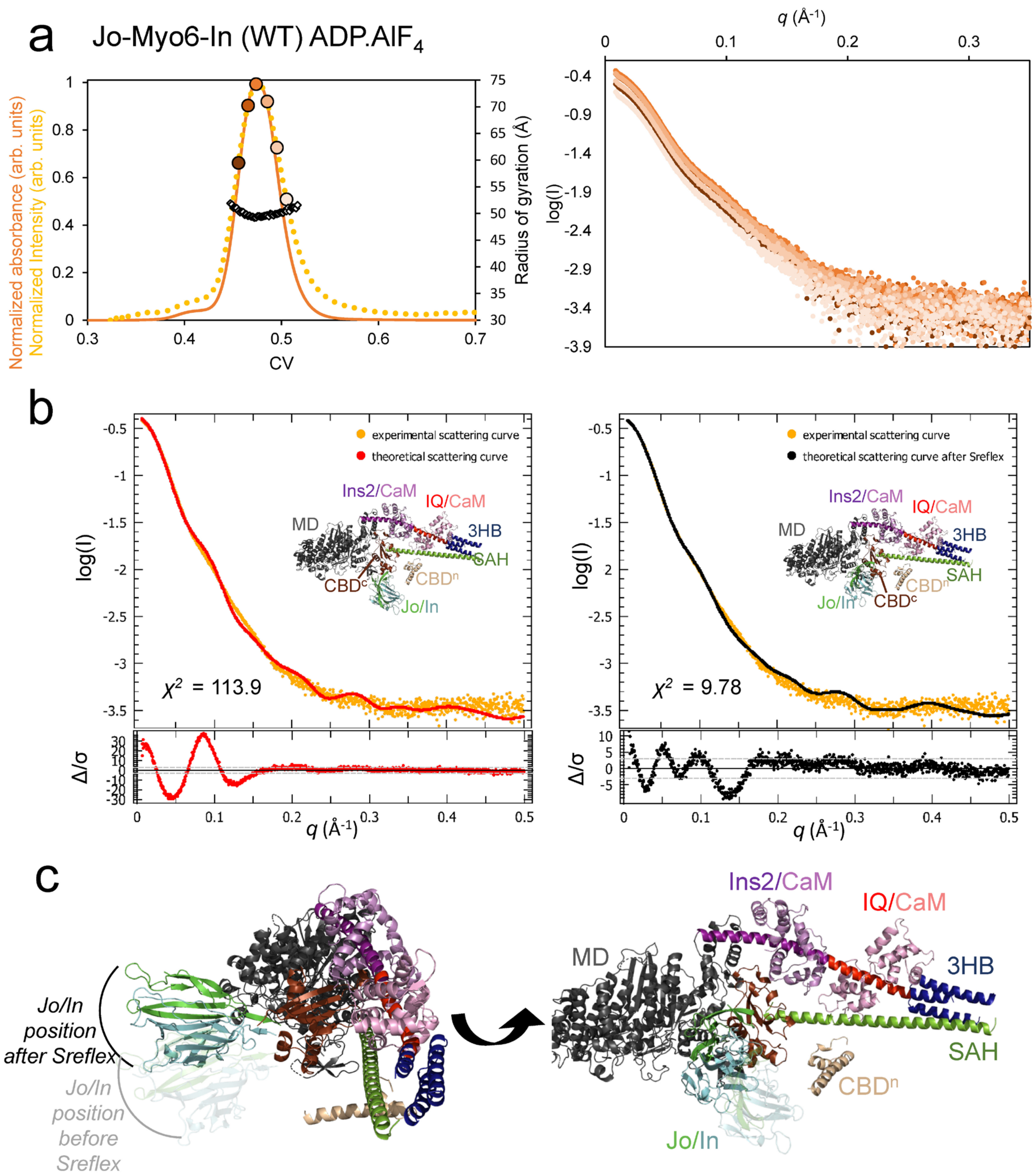

Supplementary Figure 5 – Jo-Myo6-In scattering data

(a) SEC-SAXS data for Jo-Myo6-In in ADP.AlF_4 buffer (see Methods). (Left) SEC elution profile of Jo-Myo6-In, the normalized absorbance (at 280 nm) is plotted as solid line, the normalized scattering intensity per frame is plotted as dotted line and the radius of gyration (R_g) per frame are plotted as black squares. (Right) Scattering profiles (log(I) vs q) for several frames along the peak (highlighted as colored dots).

(b) Using CRYSOL[7], the theoretical scattering curves of the Jo-Myo6-In model were generated before (red, left) and after (black, right) model refinement using SREFLEX[11]. These curves were compared to the experimental scattering curve of Jo-Myo6-In (yellow). Residuals are plotted in the bottom part of the figure. The Jo-Myo6-In model fit is improved after SREFLEX refinement.

(c) Superimposition of the Jo-Myo6-In model before and after refinement by SREFLEX. The Jo/In domain is the only part of the model to be displaced, the rest of the model remains mostly unchanged. This highlights the ability of the Jo/In domain to vibrate with respect to Myo6.

(a-b) Source data are provided as a Source Data file.

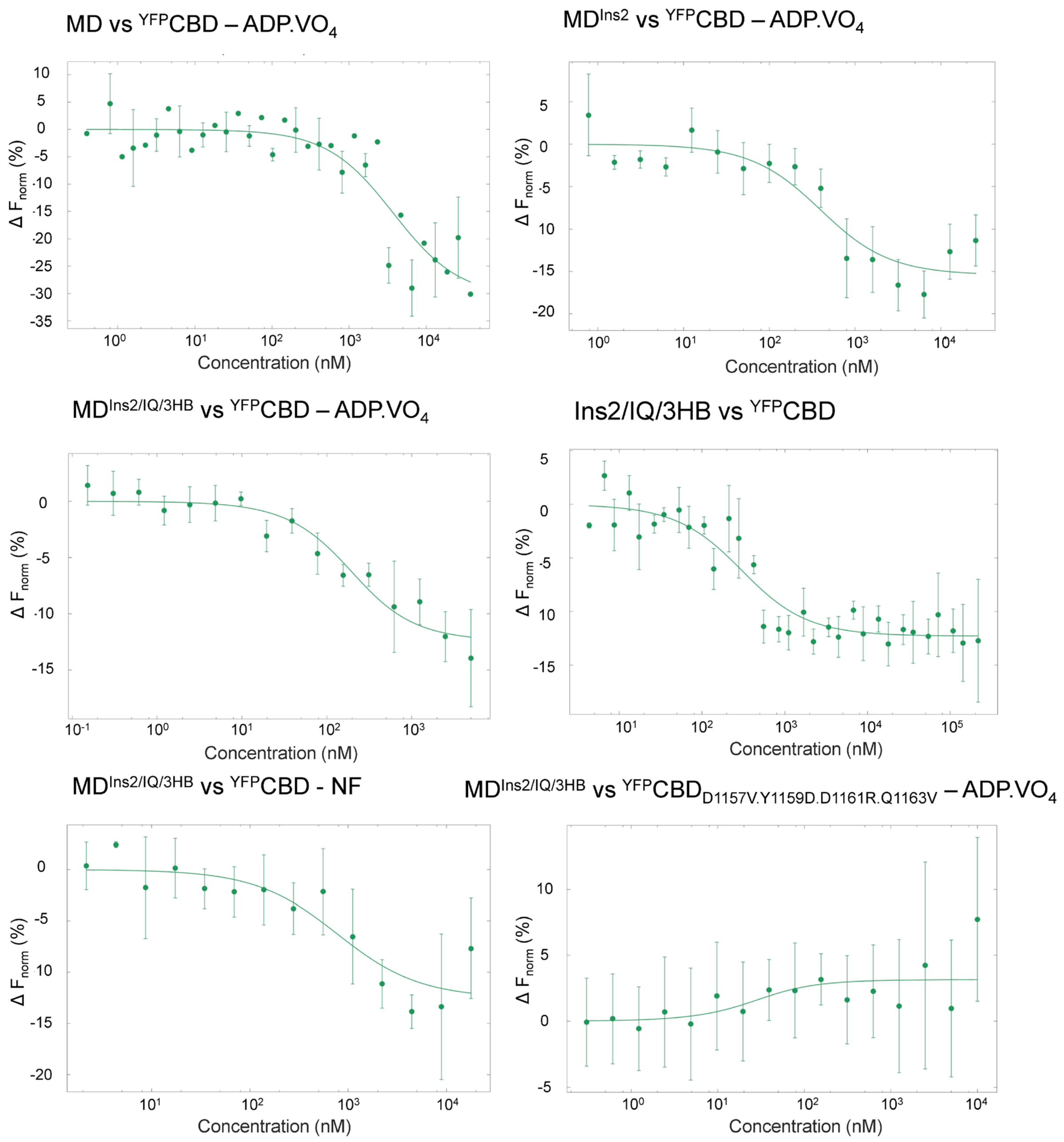

Supplementary Figure 6 – Affinity of the Myo6 CBD for different Myo6 Head or Neck constructs. Microscale thermophoresis profiles and fits corresponding to the data exposed in Table 1. Error bars = standard deviation. Data from 2-4 replicates. Microscale thermophoresis profiles and fits corresponding to the data exposed in Table 1. The baseline corrected normalized fluorescence (ΔF_{norm}) is plotted against the concentration of Myo6 Head constructs. Myo6 Tail construct was added in a constant concentration (100 nM) in all our assays (see Methods). K_D was determined with a confidence of 68 % using the NTAnalysis software. The error bars are for the standard deviation. A standard fitting model derived from the law of mass action was used:

$$F(\text{concentration}) = \text{Unbound} + \frac{(\text{Bound}-\text{Unbound})\left([\text{Myo6 Head}]+[\text{Myo6 Tail}]+K_D-\sqrt{([\text{Myo6 Head}]+[\text{Myo6 Tail}]+K_D)^2-4[\text{Myo6 Head}][\text{Myo6 Tail}]}\right)}{2[\text{Myo6 Tail}]}$$

(with Unbound corresponding to the response value of the unbound state and bound corresponding to the response value of the bound state)[12]. Source data are provided as a Source Data file.

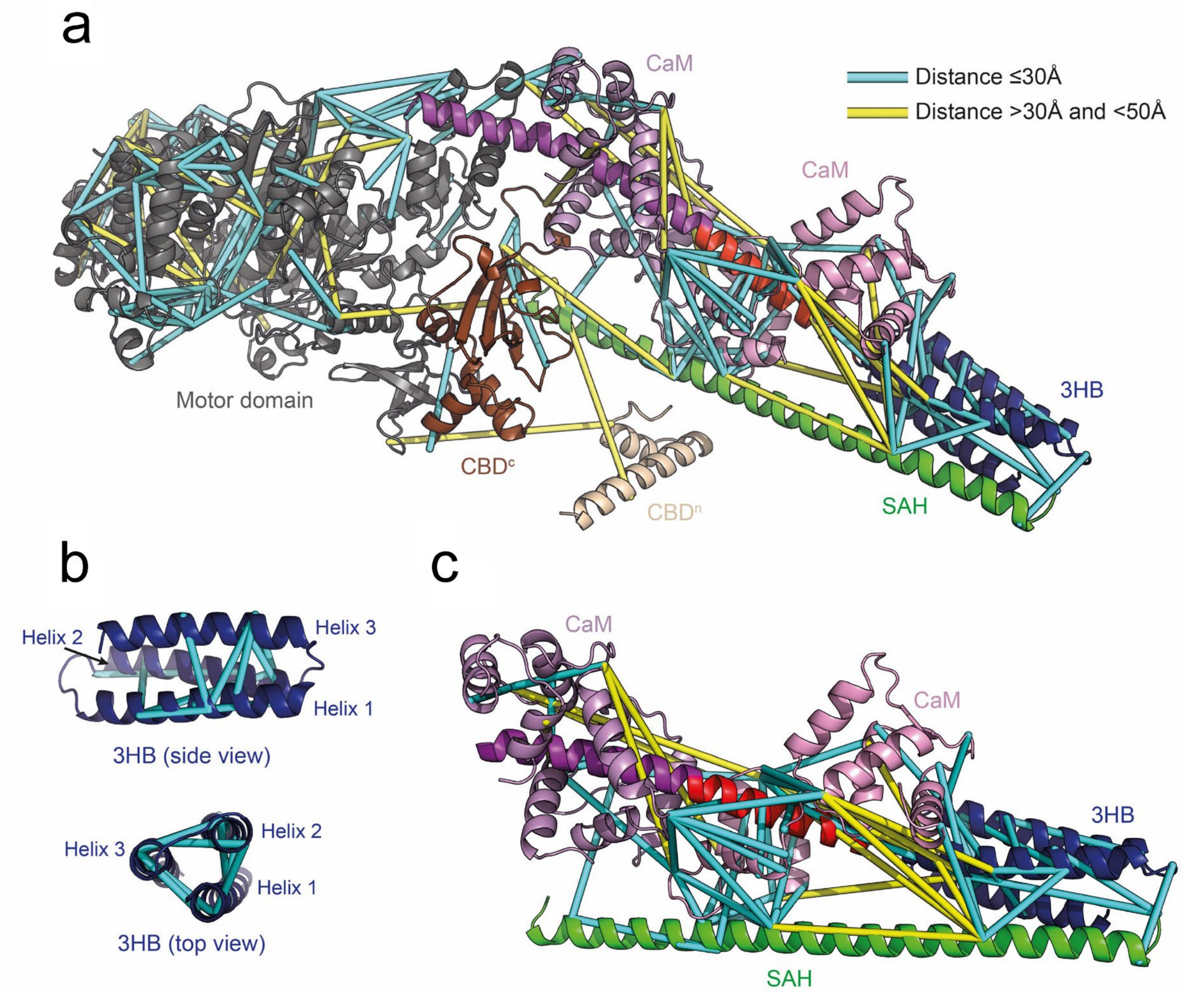

__**Supplementary Figure 7 – Crosslinking validation of the Myo6 off-state model**__

Crosslinking coupled with mass spectrometry analysis of FLMyo6 in the pre-powerstroke state using disuccinimidyl sulfoxide (DSSO) as a cross-linker (see Methods). The model is colored as follows : Motor domain (MD, grey), CaM binding sites (Ins2/IQ, purple/red), CaM (lilac/pink), 3-helix bundle (3HB, blue), single alpha helix (SAH, green), CBD^c (brown), CBD^n fragment (beige) Detected crosslinks are indicated according to their inter-Cα distance in the model: coloured lines: cyan, less or equal than 30 Å; yellow, distance greater than 30 Å. A cut-off of 50 Å was applied (see Sup Table 1 for a full-list of detected crosslinks, including violating crosslinks).

(a) Overall distribution of crosslinks. A high density of crosslinks were detected in the well-characterized motor domain (dark grey).

(b) Detected crosslinks within the 3-helix bundle.

(c) Crosslinks around the SAH helix, confirming the general position of the tail segments. Several crosslinks were detected between the SAH (green), the 3HB (dark blue) and the calmodulin light chains (light violet, light pink), confirming the negative stain model with the SAH folding back towards the motor.

Few crosslinks were also detected between the CBD^c (brown), the motor domain, the calmodulin and the SAH, further supporting the CBD^c location next to the motor domain. Finally, the CBD^n was also crosslinked with the SAH and the N-terminal SH3-like domain in the motor domain. Note that only one violating crosslinks is attributed to the CBD^c and CBD^n (see Sup Table 1), indicating that the low density of crosslinks in these domains probably reflects the high flexibility of that region, and not a problem with its placement. Indeed, the analysis was performed in the absence of the Jo-In molecule to allow the Myo6 molecule to explore a higher range of conformations.

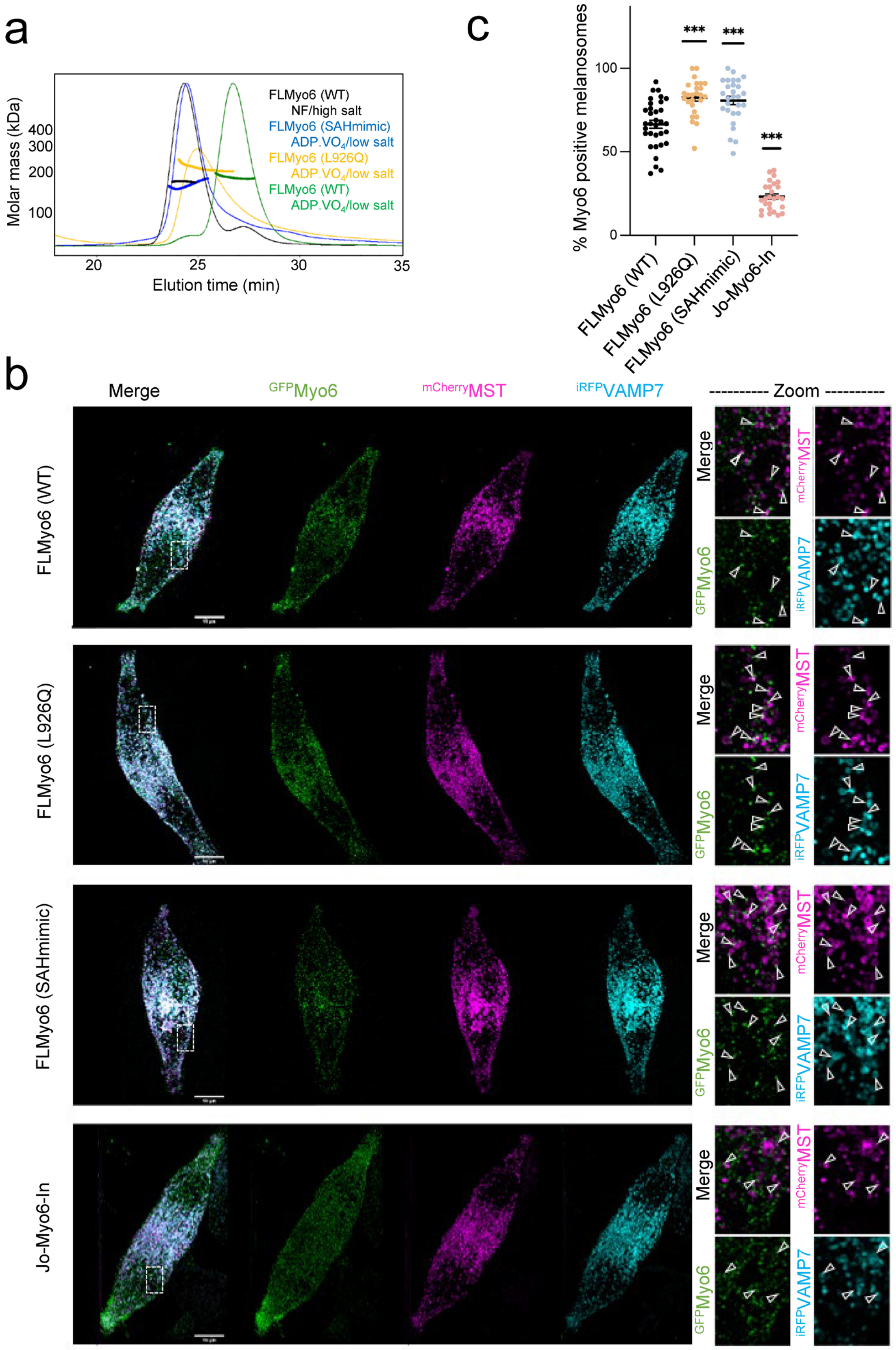

Supplementary Figure 8 – Role of the proximal Myo6 sequence in the stabilization of the off-state. **(a)** Normalized SEC-MALS profiles of FLMyo6 (SAHmimic) (blue) and FLMyo6 (L926Q) (yellow) mutants, plus FLMyo6 (WT) in conditions that favor opening (black) or closure (green), as references.

Experimental conditions were similar to those described for Sup Fig 1. Here, “High salt” means 800 mM NaCl; “low salt”, 80 mM. Source data are provided as a Source Data file.

(b) Representative fixed MNT-1 cells co-expressing ^{GFP}FLMyo6 (WT), ^{GFP}Jo-Myo6-In, ^{GFP}FLMyo6 (SAHmimic) or ^{GFP}FLMyo6 (L926Q) with ^{mCherry}MST and ^{iRFP}VAMP7. ^{mCherry}MST and ^{iRFP}VAMP7 are melanosome-associated components. MNT-1 cells were fixed 48h post-transfection, then imaged and processed for quantification. Green: ^{GFP}Myo6; Magenta: ^{mCherry}MST; Cyan: ^{iRFP}VAMP7. From left to right: Entire cell: 3 channels merge and individuals; 8x zoom of the boxed region: merged ^{GFP}Myo6 / ^{mCherry}MST, individual channels. Scale bars: 10 µm.

(c) Quantification of Myo6-positive melanosomes for ^{GFP}FLMyo6 (L926Q), ^{GFP}FLMyo6 (SAHmimic), ^{GFP}Jo-Myo6-In, and ^{GFP}FLMyo6 (WT) (~30 cells examined over 3 independent experiments). Significance: ***, $P < 0.001$ (unpaired t test with Welch’s correction). Error bars = SEM. Source data are provided as a Source Data file.

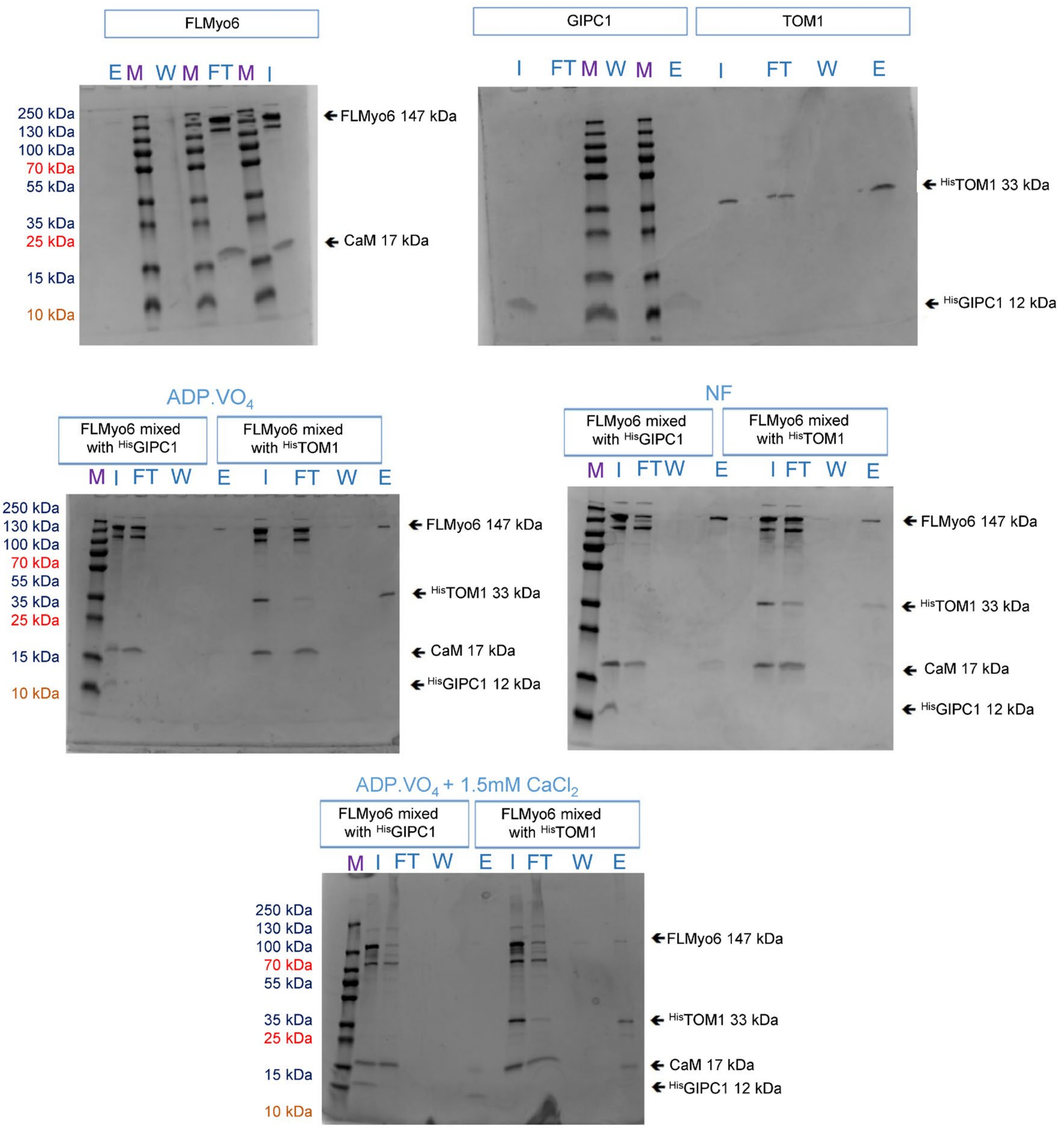

Supplementary Figure 9 – SDS-PAGE stained with colloidal blue corresponding to Anti-His pull-down in Fig. 3D.

Input (I), flow through (FT), last wash (W) and elution (E) fractions corresponding to anti-His pull-down pictured in Fig. 3D. The protein marker columns are indicated with a purple M. Uncropped and unedited gels are provided as a Source Data file.

	Partner	Myo6 Tail	K_D	n
Interaction with GIPC1	^{His}GIPC1	^{YFP}CBD (WT)	214 ± 144 nM	2
	^{His}GIPC1	^{YFP}CBD (I1072A)	227 ± 108 nM	2
Interaction with Dab2	^{His}Dab2	^{YFP}CBD (WT)	423 ± 346 nM	2
Interaction with TOM1	^{His}TOM1	^{YFP}CBD (WT)	445 ± 135 nM	2

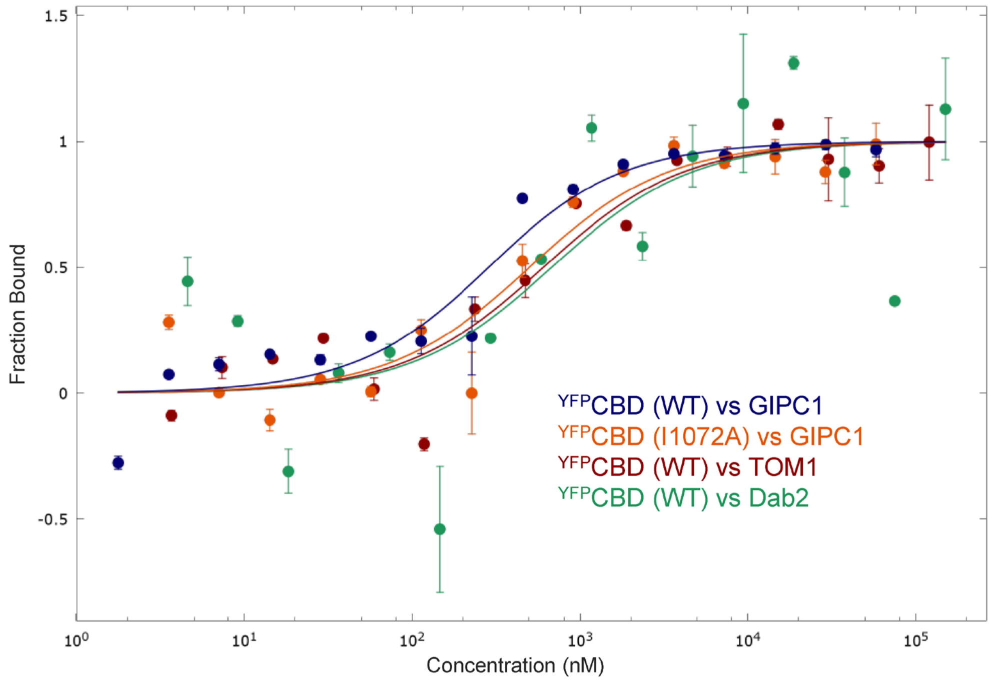

Supplementary Figure 10 – Affinity of the Myo6 tail for partners by Microscale Thermophoresis.
(Top) Dissociation constant (K_D) ± K_D confidence (with a 68% confidence using the NTAnalysis software) determined by microscale thermophoresis of Myo6 CBD constructs against Myo6 partners (n=2). **(Bottom)** Integration of thermophoresis profiles against concentration of partners (nM) for one replicate shown as an example. The affinity was quantified by analyzing the change in thermophoresis as a function of the concentration of the titrated protein using the NTAnalysis software provided by the manufacturer. Error bars: standard deviation. Fit: “Kd model” equation from NTAnalysis. Source data are provided as a Source Data file.

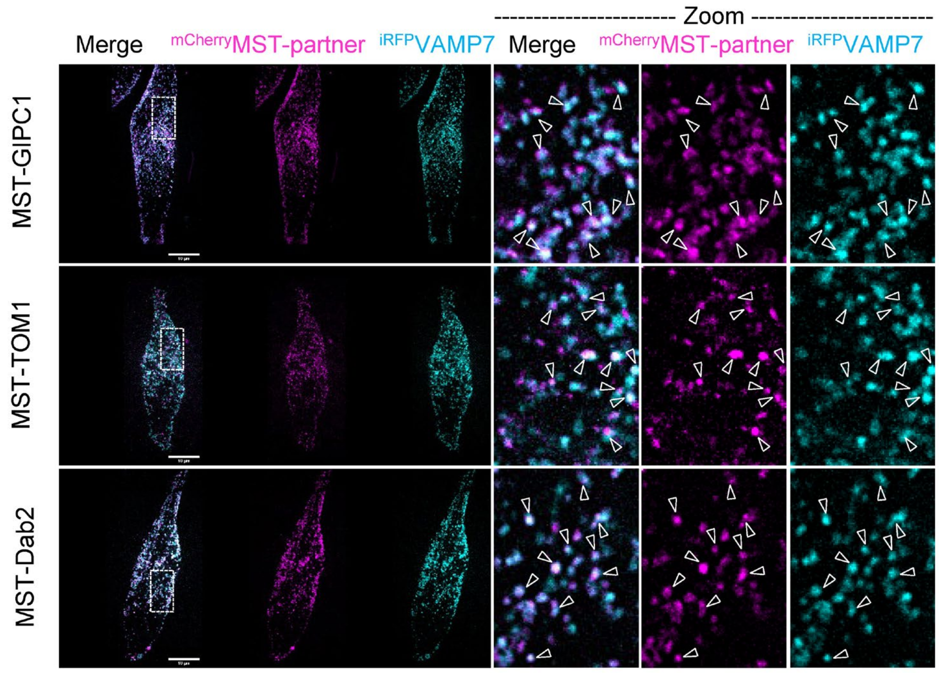

<u>Supplementary Figure 11 – Localization of MST-partners at melanosomal membrane in MNT-1 cells.</u> Representative MNT-1 cells co-expressing ^{mCherry}MST-partners and ^{iRFP}VAMP7. MNT-1 were fixed 48h post-transfection then imaged. In order: ^{mCherry}MST-GIPC1, ^{mCherry}MST-TOM1, ^{mCherry}MST-Dab2. Magenta: ^{mCherry}MST-partners; Cyan: ^{iRFP}VAMP7. From left to right: entire cell (3 channels merged, individual channels); zoom 8x boxed region (^{mCherry}MST-partners / ^{iRFP}VAMP7 merged, individual channels). Results show colocalization of ^{mCherry}MST-partners all around the ^{iRFP}VAMP7 melanosomes.

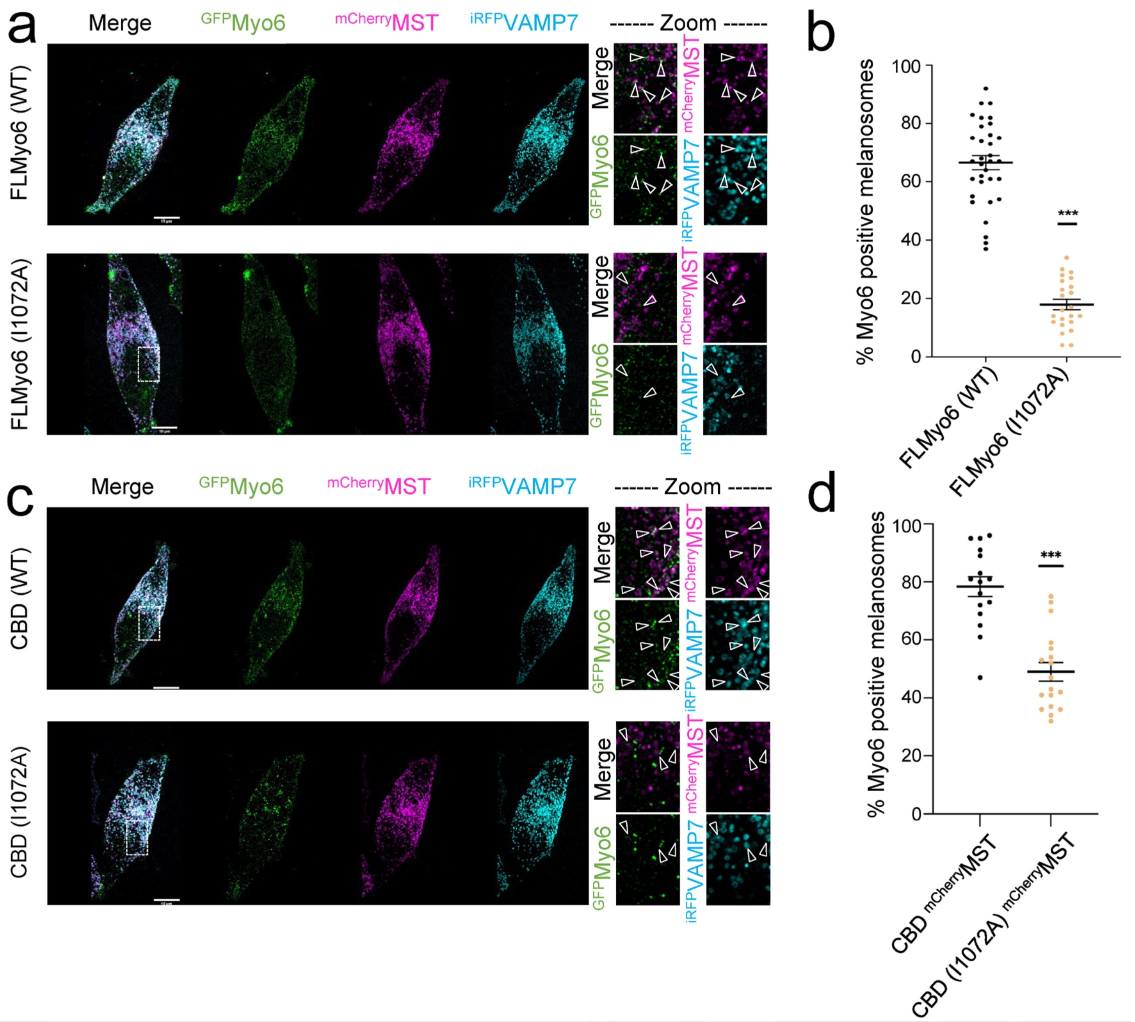

Supplementary Figure 12 – I1072 is key for Myo6 specific recruitment on melanosomes.

(a) Representative fixed MNT-1 cells co-expressing ^{GFP}Myo6 +/- I1072A mutation with ^{mcherry}MST and ^{iRFP}VAMP7. ^{mCherry}MST and ^{iRFP}VAMP7 are melanosome-associated components. MNT-1 cells were fixed 48h post-transfection then imaged and processed for quantification. Note that the control FLMyo6 (WT) is the same as in Sup Fig. 8B.

(b) Quantification of Myo6-positive melanosomes for ^{GFP}FLMyo6 (WT) and ^{GFP}FLMyo6 (I1072A) in MNT-1 cells (~30 cells examined over 3 independent experiments). Source data are provided as a Source Data file.

(c) Representative MNT-1 cells co-expressing Myo6 CBD +/- I1072A mutation with ^{mcherry}MST and ^{iRFP}VAMP7. ^{mCherry}MST and ^{iRFP}VAMP7 are melanosome-associated components. MNT-1 cells were fixed 48h post-transfection then imaged and processed for quantification.

(d) Quantification of Myo6-positive melanosomes for ^{GFP}CBD (WT) and ^{GFP}CBD (I1072A) in MNT-1 cells (~20 cells examined over 2 independent experiments). Source data are provided as a Source Data file.

(a, c) Green: ^{GFP}Myo6 constructs; Cyan: ^{iRFP}VAMP7; Magenta: ^{mcherry}MST-partner. Entire cell merge 3 channels and individuals. Zoom (8x boxed region): merge ^{GFP}Myo6 / ^{mcherry}MST-partner, individual channels. Scale bars: 10µm.

(b, d) Myo6-positive melanosomes are expressed in percentage and normalized to the total number of VAMP7-positive melanosomes. Significance: ***, $P < 0.001$ (unpaired t test with Welch's correction), for each ^{GFP}Myo6 construct, significance of experiments with partners compared to the control without partner (in black on the graph). Error bars = SEM.

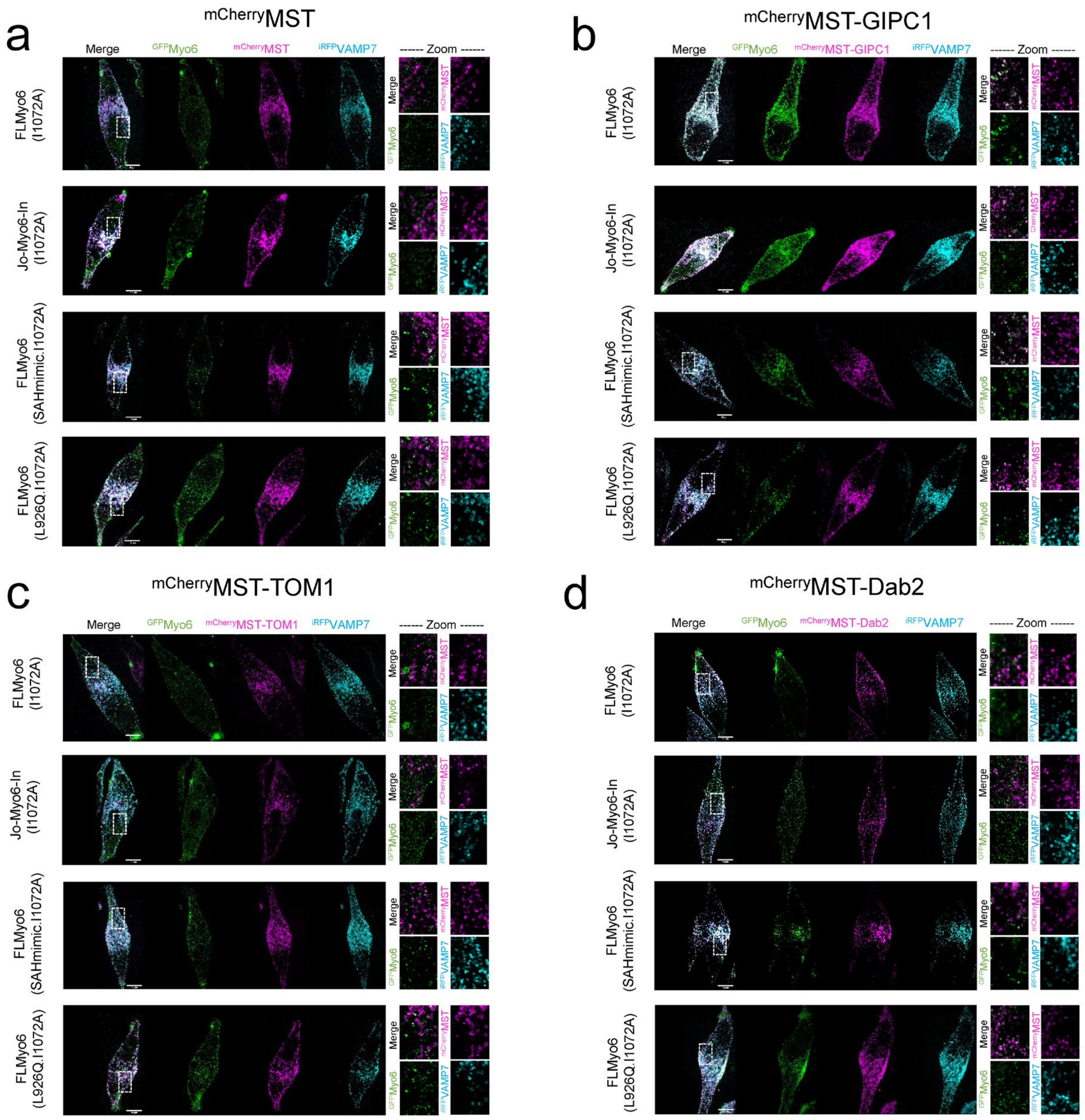

Supplementary Figure 13 – GIPC1 can bind the back-folded Myo6 state and activate it, while Dab2 and Tom1 can only bind to Myo6 once the motor has been primed open.

Representative entire MNT-1 cells corresponding to the zooms in Fig. 4. Left to right: merge of 3 channels, individual channels. Zoom (8x boxed region): merge ^{GFP}Myo6 / ^{mCherry}MST-partners, individual channels. Scale bars: 10µm. ^{GFP}FLMyo6 constructs: ^{GFP}FLMyo6 (I1072A), ^{GFP}FLMyo6 (SAHmimic.I1072A), ^{GFP}FLMyo6 (L926Q.I1072A), Jo-Myo6-In (I1072A).

(a) Representative fixed MNT-1 cells co-expressing different ^{GFP}FLMyo6 (I1072A) constructs with control ^{mCherry}MST and ^{iRFP}VAMP7.

(b) Representative fixed MNT-1 cells co-expressing different ^{GFP}FLMyo6 (I1072A) constructs with ^{mCherry}MST-GIPC1 and ^{iRFP}VAMP7.

(c) Representative fixed MNT-1 cells co-expressing different ^{GFP}FLMyo6 (I1072A) constructs with ^{mCherry}MST-TOM1 and ^{iRFP}VAMP7.

(d) Representative fixed MNT-1 cells co-expressing different ^{GFP}FLMyo6 (I1072A) constructs with ^{mCherry}MST-Dab2 and ^{iRFP}VAMP7.

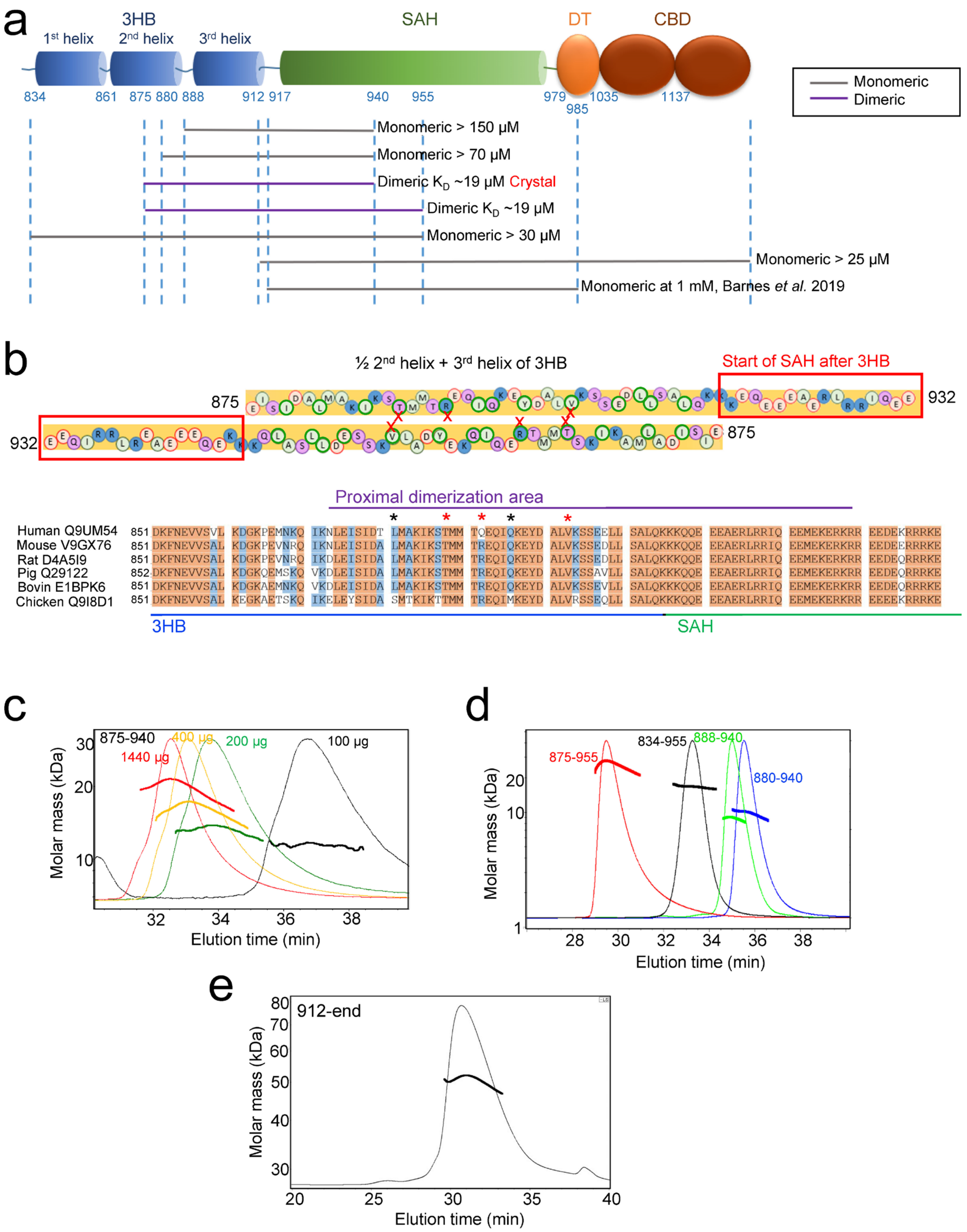

Supplementary Figure 14 –Characterization of the dimerization of proximal region by MALS.

(a) Scheme of the Myo6 Tail showing the oligomerization states found for different constructs using SEC-MALS in 10 mM Tris pH 7.5; 50 mM NaCl; 5 mM NaN_3; 0.5 mM TCEP.

The 875-940 fragment is sufficient for dimerization. Importantly, a peptide missing the first 5 aa (aa 880-940) failed to dimerize even when its peak concentration was higher than 70 μM, which emphasised the key role of residues 875-880 for the stability of the dimerization. Additionally, in line with a recent study[13], no dimerization was found via the SAH, unlike previously proposed by molecular

dynamics simulations[14]. Indeed, no dimerization was observed for the Tail fragment aa 912-end, (containing the whole SAH), even when its peak concentration was 25 µM.

(b) (Top) schematic representation of the dimer crystal structure. Negatively charged residues are circled in red, positively charged residues are circled in blue, hydrophobic residues are circled in green and polar, uncharged residues are circled in purple. Residues involved in apolar contacts are circled in green. Three residues found to be essential for dimerization (T888D, R892E, V903D) are marked with a red cross. **(Bottom)** Sequence alignment of 851-949 (human protein numbering). Red: conserved residues; blue: conserved in 5 out of 6 species. Black star: residues inside the dimerization area that are not 100% conserved (S881 and M896). In the structure, M896 faces itself and seems compatible with the crystallized dimer. Red star: residues mutated in this study to disrupt proximal dimerization. Residues T888 and V903 are well conserved. The crystal structure shows that the replacement of R892 by Q in the human sequence should be compatible with proximal dimerization.

(c) Titration of the 875-940 fragment by SEC-MALS in 10 mM Tris pH 7.5; 50 mM NaCl; 5 mM NaN_3; 0.5 mM TCEP. Thin lines: light scattering (normalized); thick lines: molecular masses. A K_D^{App} of ~19 µM was calculated from a fit of molecular weight against the concentration at the peak from each profile – the affinity is probably underestimated because of the size exclusion resin.

(d) Normalized SEC-MALS profiles of the 834-955, 880-940 and 888-940 constructs (concentration at the peak stated on Sup Fig. 14A) found to be monomeric in 10 mM Tris pH 7.5; 50 mM NaCl; 5 mM NaN_3; 0.5 mM TCEP, unlike the dimeric 875-955 construct (red).

(e) Normalized SEC-MALS profile of the 912-end construct (25 µM at the peak) found to be monomeric in 10 mM Tris pH 7.5; 50 mM NaCl; 5 mM NaN_3; 0.5 mM TCEP.

(c-e) Source data are provided as a Source Data file.

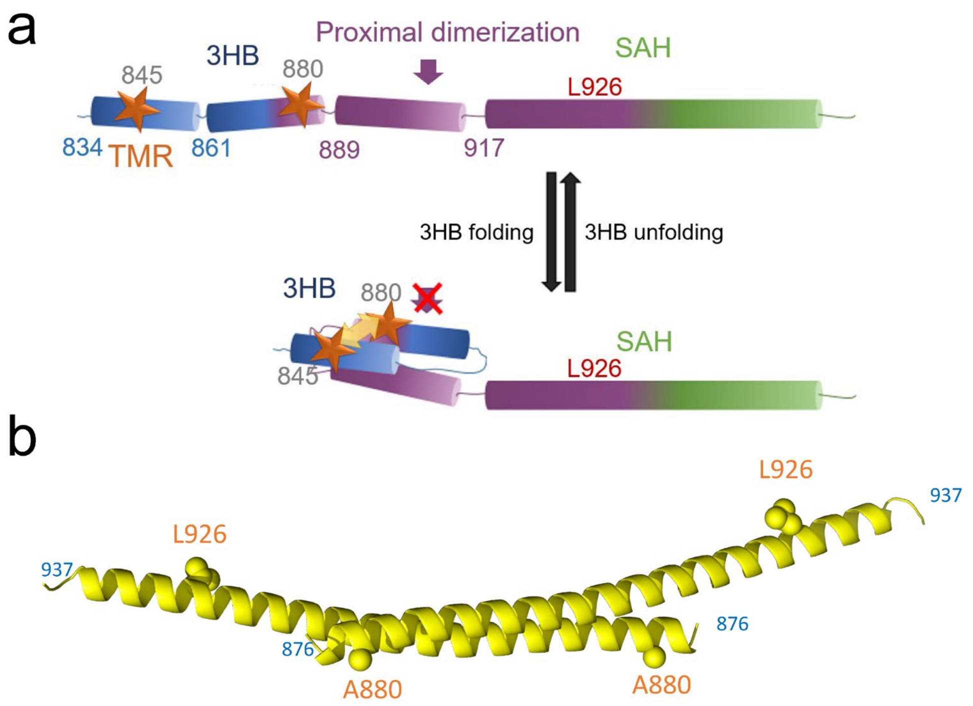

Supplementary Figure 15 – Proximal dimerization is regulated by 3HB unfolding which is impaired by the pathogenic L926Q deafness mutation.

(a) Scheme representing Myo6 SAH (green) and 3HB (blue), with the proximal dimerization area (875-940) shown in purple. Stars: TMR fluorescent spots. Their proximity promotes quenching when the 3HB is folded.

(b) Crystal structure of the proximal dimer pictured in cartoon, residues A880 and L926 are shown as spheres.

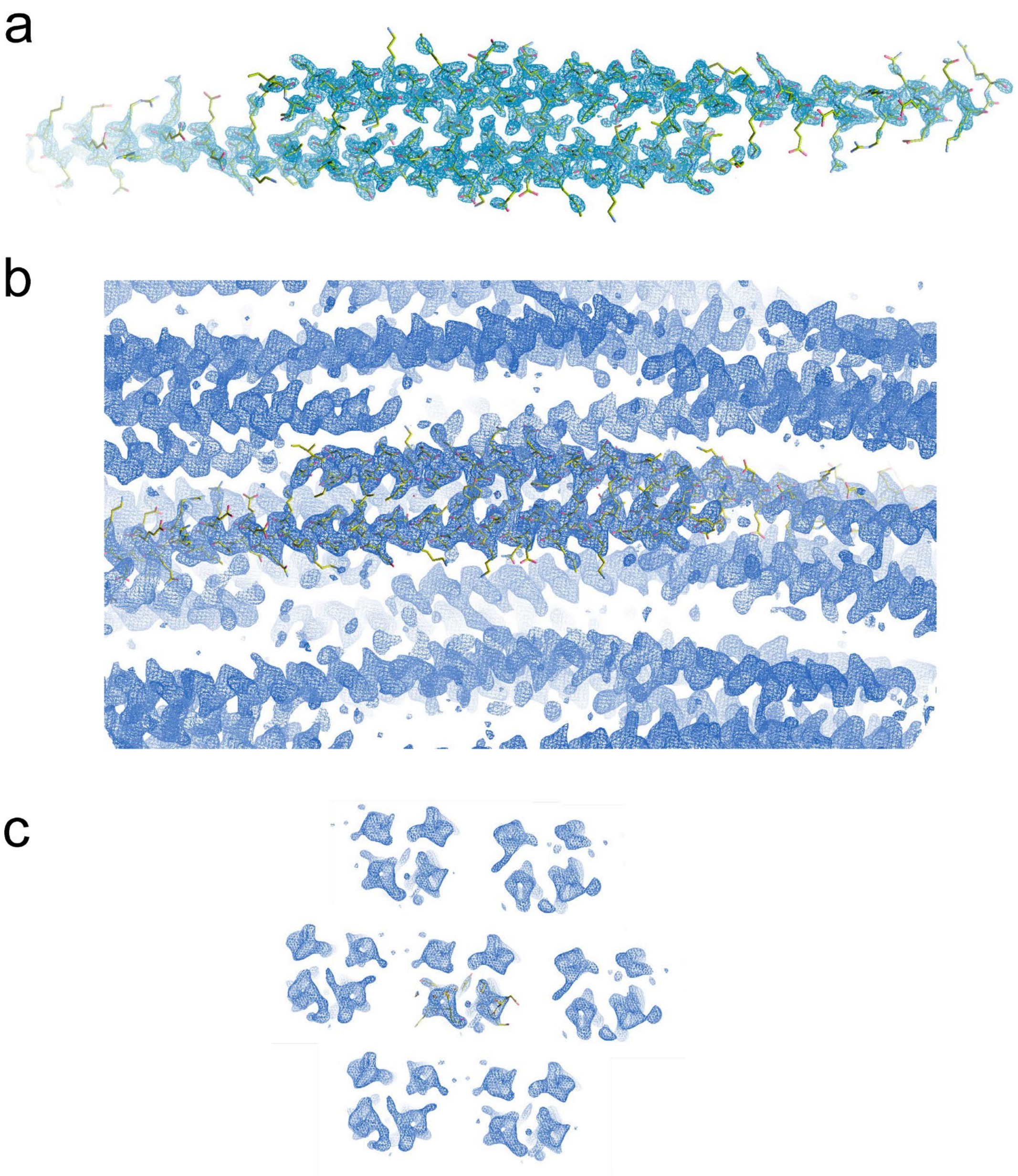

<u>Supplementary Figure 16 – Electron density of the Myo6 875-940 crystal structure.</u>

(a) Electron density corresponding to the 875-940 dimer (2Fo-Fc map contoured at 1.0 RMSD).

(b) View of the 2-fold axis that defines the antiparallel dimer.

(c) View of the 6-fold axis. Although a high number of copies can be seen in the asymmetric unit, the 875-937 helix makes extensive contacts over 11.5 turns with another helix. The rest of the crystal contacts made by the helix are negligible in comparison (Sup Movie 2). Images prepared with Coot 0.9.8.1[15].

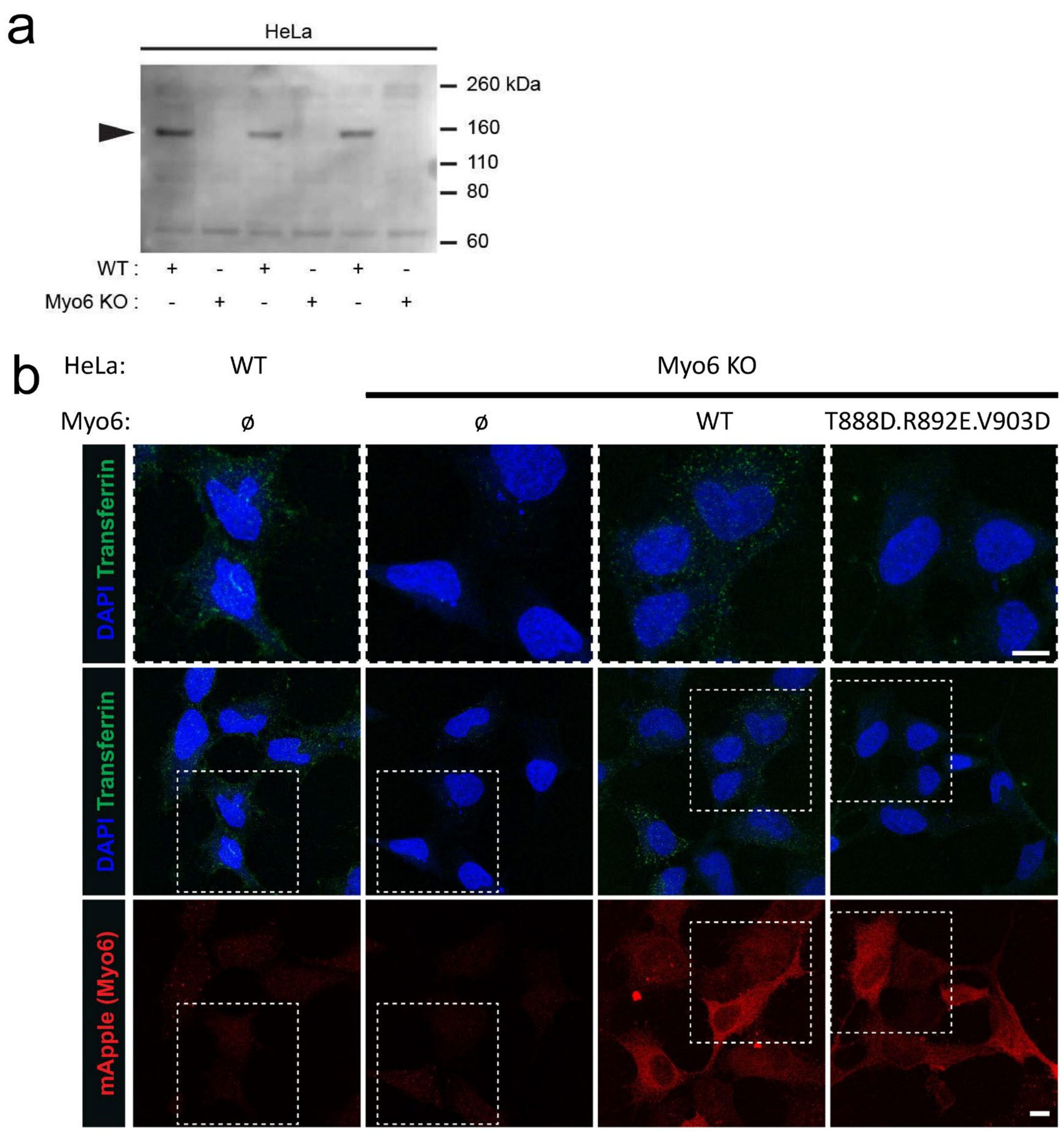

<u>Supplementary Figure 17 – Endocytosis is impaired if Myo6 cannot dimerize through its proximal region</u>

(a) Confirmation of Myo6 null HeLa cells by western blot. Uncropped and unedited gel is provided as a Source Data file.

(b) Endocytic uptake of fluorescently-labeled transferrin by WT and Myo6 null HeLa cells. Internalized transferrin appears in green, HeLa cell nuclei (DAPI) in blue, and expression of ^{mApple}Myo6 constructs in red. Representative maximum-intensity projection images demonstrate a marked reduction in internalized transferrin in Myo6 mutant HeLa cells compared to wild-type cells. Forced expression of FLMyo6 (WT), but not of FLMyo6 (T888D.R892E.V903D, triple mutant), restores the ability of Myo6 null HeLa cells to uptake transferrin. Scale bar: 10 µm. Source data are provided as a Source Data file.

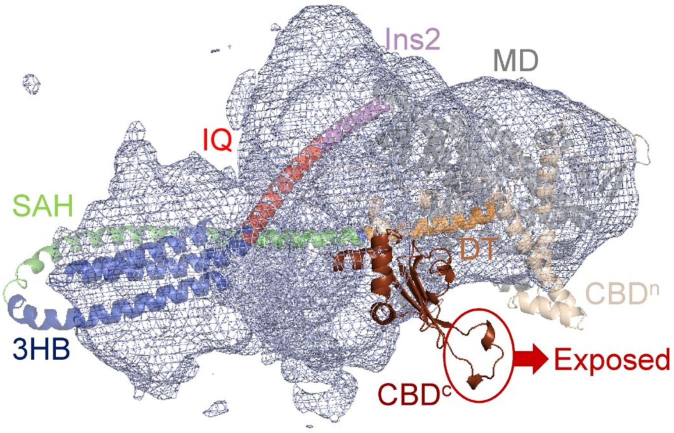

Supplementary Figure 18 – Alphafold[16] predicts a back-folded FLMyo6 with an exposed CBD^c

Alphafold modelisation of FLMyo6 (UNIPROT: Q9UM54) was manually docked within our 3D reconstruction of Jo-Myo6-In (grey mesh). CBD^c is found outside of the electron density, with residues D1157, Y1159, D1161, Q1163 highly exposed (red circle), inconsistently with our experimental data.

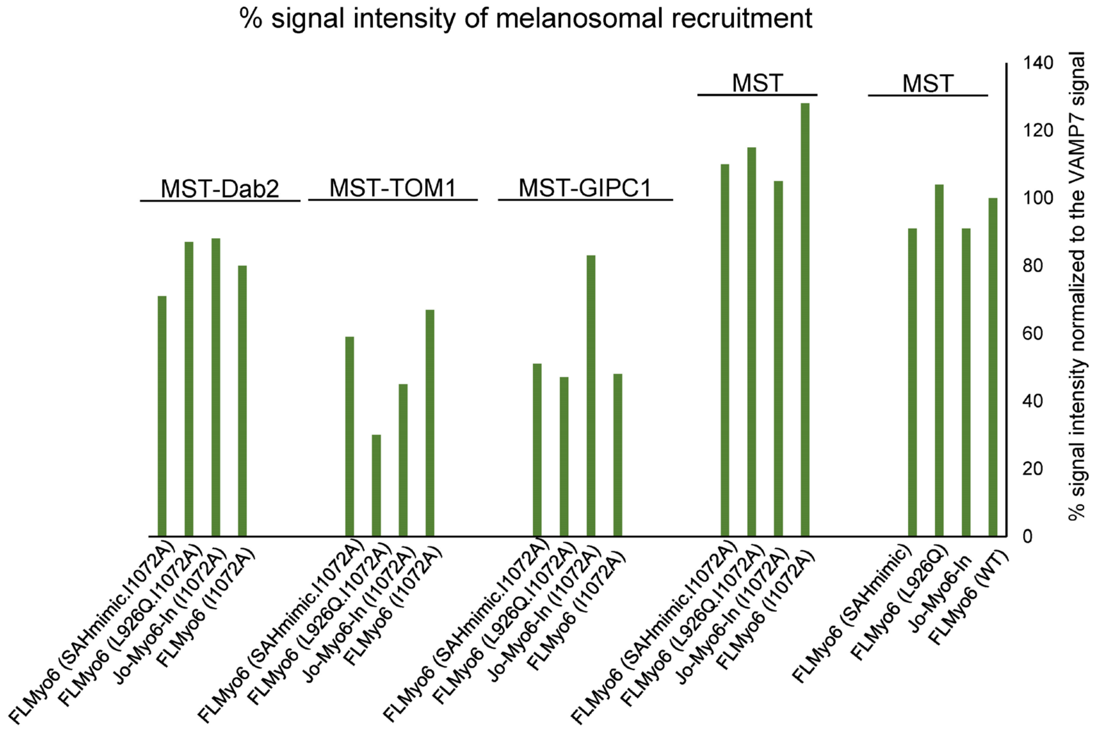

Supplementary Figure 19 – Fluorescence of MST-partners localized on melanosomes, normalized to the VAMP7 signal.

Quantification of the fluorescence signal of ^{mCherry}MST-partners on melanosomes in MNT-1 cells expressing different Myo6 constructs. Global intensities were calculated by using the macro described in Methods, normalized to the ^{iRFP}VAMP7 signal (total melanosomal surface area). Source data are provided as a Source Data file.

Supplementary Text for crosslinking mass spectrometry

To further validate our structural model, we carried out a crosslinking mass spectrometry (XL-MS) analysis of the purified FLMyo6 (Sup Fig. 7A-C, Sup Table 1). 213 out of 247 detected crosslinks are in agreement with the structural model since they correspond to distance between Cα < 50 Å, with 171 crosslinks having a distance less than 30 Å. The SAH can be detected in close proximity of the 3HB and the CaM light chains, further confirming the folding back of the molecule (Fig. 3A, Sup Fig. 7C). Interestingly, a relatively high number of crosslinks were detected between the helices in the 3-helix bundle (Sup Fig. 7B), confirming that the bundle remains closed in the off-state.

Overall, only a few crosslinks were detected within the CBD region and the rest of the molecule. One crosslink was found between the CBD^c and the Motor and one crosslink was identified between the CBD^c and the SAH. Taken together, these contacts situate the CBD^c between the Motor, CaM and SAH as expected in our model.

Finally, two crosslinks were detected between the CBD^n and the rest of the Myo6. Both crosslinks are in agreement with our model. Note that overall, only two crosslinks were detected for the CBD^n region, even when considering detected distances >50 Å. These results suggest a high flexibility in this region. Interestingly, this flexibility could allow for a higher accessibility of the RRL motif, further confirming our model shown in Fig. 3A.

Together, our data suggests a model in which the tail of Myo6 folds back towards the 3HB, with the SAH in close proximity to the neck region, and the CBD^c nested in a groove between the Lever arm and the Motor only accessible when the molecule is in the pre-powerstroke state.

Supplementary Tables

Protein 1	Residue 1	Protein 2	Residue 2	Distance (Å)	Protein 1	Residue 1	Protein 2	Residue 2	Distance (Å)	Protein 1	Residue 1	Protein 2	Residue 2	Distance (Å)
Myo6	39	Myo6	1058	41.2	Myo6	325	Myo6	330	6.4	Myo6	845	CaM	145	31.3
Myo6	78	Myo6	82	6.3	Myo6	334	Myo6	917	169.0	Myo6	845	Myo6	897	16.1
Myo6	81	Myo6	105	16.7	Myo6	334	Myo6	935	141.9	Myo6	845	Myo6	852	11.2
Myo6	82	Myo6	196	29.0	Myo6	334	CaM	78	97.2	Myo6	845	Myo6	904	14.7
Myo6	83	Myo6	657	38.4	Myo6	334	Myo6	963	102.9	Myo6	847	Myo6	879	8.3
Myo6	83	Myo6	1189	35.7	Myo6	334	Myo6	402	44.0	Myo6	851	Myo6	917	18.5
Myo6	83	CaM	183	59.3	Myo6	334	CaM	116	82.1	Myo6	852	Myo6	935	16.4
Myo6	83	Myo6	105	14.8	Myo6	334	Myo6	917	169.0	Myo6	852	Myo6	884	23.5
Myo6	102	Myo6	307	17.0	Myo6	334	Myo6	935	141.9	Myo6	852	CaM	119	50.6
Myo6	113	Myo6	170	8.8	Myo6	334	Myo6	963	102.9	Myo6	852	Myo6	869	15.4
Myo6	118	CaM	31	54.5	Myo6	334	CaM	178	88.3	Myo6	852	Myo6	935	16.4
Myo6	118	Myo6	771	11.7	Myo6	334	Myo6	852	155.1	Myo6	852	Myo6	884	23.5
Myo6	122	Myo6	771	14.8	Myo6	365	Myo6	408	20.3	Myo6	861	Myo6	920	15.9
Myo6	139	Myo6	153	33.1	Myo6	365	Myo6	405	30.0	Myo6	864	Myo6	880	23.6
Myo6	139	Myo6	719	21.6	Myo6	402	Myo6	634	13.8	Myo6	884	Myo6	897	13.2
Myo6	139	CaM	31	57.3	Myo6	402	Myo6	412	30.4	Myo6	884	Myo6	898	10.4
Myo6	139	Myo6	217	8.8	Myo6	402	Myo6	634	13.8	Myo6	884	Myo6	897	13.2
Myo6	139	Myo6	771	12.2	Myo6	402	Myo6	412	30.4	Myo6	886	CaM	30	17.4
Myo6	139	Myo6	893	106.4	Myo6	403	Myo6	414	31.2	Myo6	886	CaM	25	14.2
Myo6	139	Myo6	771	12.2	Myo6	403	Myo6	984	95.3	Myo6	886	Myo6	898	14.1
Myo6	139	Myo6	217	8.8	Myo6	403	Myo6	573	49.3	Myo6	886	CaM	97	17.4
Myo6	142	Myo6	720	21.3	Myo6	403	CaM	132	107.1	Myo6	935	Myo6	957	33.1
Myo6	142	Myo6	634	45.8	Myo6	403	Myo6	625	21.4	Myo6	935	CaM	116	45.0
Myo6	142	Myo6	719	21.5	Myo6	408	Myo6	634	18.9	Myo6	935	CaM	78	33.6
Myo6	161	Myo6	196	19.3	Myo6	408	Myo6	625	23.9	Myo6	935	CaM	7	20.8
Myo6	162	Myo6	615	34.5	Myo6	408	Myo6	637	14.5	Myo6	935	CaM	162	50.0
Myo6	162	Myo6	657	21.5	Myo6	408	Myo6	414	17.3	Myo6	935	CaM	98	36.6
Myo6	162	Myo6	197	21.0	Myo6	408	Myo6	634	18.9	Myo6	945	Myo6	1262	66.2
Myo6	162	Myo6	177	16.8	Myo6	408	Myo6	629	26.5	Myo6	957	CaM	78	27.0
Myo6	162	Myo6	657	21.5	Myo6	412	CaM	78	115.2	Myo6	957	CaM	120	24.3
Myo6	162	Myo6	197	21.0	Myo6	412	Myo6	425	19.7	Myo6	957	CaM	128	13.2
Myo6	177	Myo6	326	9.1	Myo6	425	Myo6	615	19.9	Myo6	957	CaM	140	4.5
Myo6	177	Myo6	334	20.6	Myo6	486	Myo6	490	4.8	Myo6	957	CaM	141	2.7
Myo6	180	Myo6	326	8.7	Myo6	490	Myo6	499	14.1	Myo6	957	CaM	134	16.2
Myo6	184	Myo6	326	7.2	Myo6	505	Myo6	750	16.9	Myo6	957	CaM	145	27.0
Myo6	184	Myo6	334	15.5	Myo6	606	Myo6	629	16.6	Myo6	957	CaM	162	18.2
Myo6	196	Myo6	287	19.0	Myo6	606	Myo6	637	13.9	Myo6	963	CaM	95	14.2
Myo6	196	Myo6	307	12.2	Myo6	615	CaM	78	106.1	Myo6	963	CaM	111	26.6
Myo6	196	Myo6	408	40.7	Myo6	615	CaM	95	102.1	Myo6	963	Myo6	967	6.1
Myo6	196	Myo6	208	17.6	Myo6	615	CaM	31	100.2	Myo6	963	CaM	178	26.6
Myo6	196	CaM	102	85.4	Myo6	615	Myo6	648	32.7	Myo6	963	Myo6	1167	39.0
Myo6	196	Myo6	228	18.7	Myo6	615	Myo6	852	164.4	Myo6	963	Myo6	979	24.7
Myo6	196	Myo6	308	11.1	Myo6	615	Myo6	648	32.7	Myo6	965	CaM	95	9.6
Myo6	196	Myo6	289	22.3	Myo6	629	Myo6	637	17.6	Myo6	965	CaM	134	11.9
Myo6	197	Myo6	240	9.8	Myo6	629	Myo6	657	27.2	Myo6	965	CaM	128	18.2
Myo6	197	Myo6	296	20.7	Myo6	629	Myo6	656	27.2	Myo6	965	CaM	169	9.3
Myo6	197	Myo6	412	32.7	Myo6	629	Myo6	657	27.2	Myo6	979	CaM	31	28.2
Myo6	197	Myo6	725	69.2	Myo6	631	Myo6	646	18.4	Myo6	984	Myo6	1087	41.7
Myo6	197	Myo6	615	34.7	Myo6	634	Myo6	646	19.1	Myo6	1160	CaM	140	32.6

Myo6	197	Myo6	307	14.2	Myo6	634	Myo6	887	145.8	Myo6	1165	Myo6	1189	15.1
Myo6	197	CaM	116	76.7	Myo6	637	Myo6	648	23.6	Myo6	1165	Myo6	1167	6.2
Myo6	197	Myo6	240	9.8	Myo6	646	Myo6	657	17.0	Myo6	1167	Myo6	1189	12.1
Myo6	197	Myo6	296	20.7	Myo6	646	Myo6	656	15.8	Myo6	1189	Myo6	1199	18.5
Myo6	197	Myo6	262	30.5	Myo6	710	Myo6	725	22.5	Myo6	1231	Myo6	1262	18.9
Myo6	197	Myo6	615	34.7	Myo6	720	Myo6	772	11.2	CaM	18	CaM	31	14.4
Myo6	197	Myo6	307	14.2	Myo6	725	CaM	30	34.5	CaM	21	CaM	95	24.0
Myo6	197	Myo6	208	18.2	Myo6	725	CaM	116	11.8	CaM	22	CaM	111	14.9
Myo6	217	Myo6	720	23.9	Myo6	725	CaM	119	11.4	CaM	22	CaM	32	8.6
Myo6	217	Myo6	771	17.8	Myo6	725	CaM	183	11.8	CaM	22	CaM	140	30.3
Myo6	217	Myo6	657	23.0	Myo6	725	CaM	97	34.5	CaM	30	CaM	116	21.4
Myo6	226	Myo6	771	34.5	Myo6	819	CaM	84	12.0	CaM	31	CaM	116	18.4
Myo6	233	Myo6	240	14.3	Myo6	822	Myo6	830	12.2	CaM	78	CaM	95	20.4
Myo6	240	Myo6	302	15.9	Myo6	822	CaM	116	9.7	CaM	78	CaM	111	28.0
Myo6	240	Myo6	307	17.3	Myo6	822	CaM	78	17.2	CaM	78	CaM	82	18.3
Myo6	262	Myo6	287	23.6	Myo6	822	CaM	81	11.2	CaM	78	CaM	78	22.5
Myo6	262	Myo6	615	17.9	Myo6	822	CaM	79	14.5	CaM	78	CaM	115	22.5
Myo6	262	Myo6	402	45.2	Myo6	822	CaM	145	17.2	CaM	78	CaM	83	19.7
Myo6	262	Myo6	334	13.3	Myo6	830	Myo6	842	18.1	CaM	95	CaM	95	35.5
Myo6	285	CaM	83	86.1	Myo6	830	CaM	183	19.0	CaM	95	CaM	111	14.0
Myo6	287	Myo6	307	11.5	Myo6	842	Myo6	917	29.7	CaM	95	CaM	105	37.9
Myo6	287	CaM	116	85.6	Myo6	842	CaM	78	30.4	CaM	97	CaM	183	21.4
Myo6	287	Myo6	334	22.9	Myo6	842	Myo6	897	12.3	CaM	98	CaM	183	18.4
Myo6	287	CaM	145	91.7	Myo6	842	CaM	21	17.9	CaM	116	CaM	116	42.6
Myo6	287	Myo6	307	11.5	Myo6	842	CaM	130	32.9	CaM	116	CaM	119	48.4
Myo6	289	Myo6	858	159.4	Myo6	842	Myo6	935	12.0	CaM	116	CaM	130	20.4
Myo6	289	CaM	134	91.1	Myo6	842	CaM	183	37.0	CaM	116	CaM	128	18.2
Myo6	289	Myo6	305	10.7	Myo6	845	Myo6	886	15.9	CaM	145	CaM	162	20.4
Myo6	296	Myo6	307	15.6	Myo6	845	CaM	95	50.4	CaM	145	CaM	183	25.7
Myo6	307	CaM	100	87.2	Myo6	845	CaM	116	40.7	CaM	145	CaM	145	22.5
Myo6	325	Myo6	334	9.0	Myo6	845	Myo6	852	11.2	CaM	162	CaM	183	20.2

Supplementary Table 1 – Detected crosslinks

Detected crosslinks within the FLMyo6 protein in the ATP state using disuccinimidyl sulfoxide (DSSO) as a cross-linker (see Methods and Sup Fig. 7). The distances (Å) between the crosslinked residues correspond to the distance in the model (see Fig. 4A): less or equal than 30 Å (171 crosslinks from a total of 247); distance greater than 30 Å but smaller than 50 Å (42/247), distance greater than 50 Å (violating crosslinks, 34/247).

Diffraction limits (Å) and corresponding principal axes of the ellipsoid fitted to the diffraction cut-off surface as direction cosines in the orthogonal basis (standard PDB convention), and in terms of reciprocal unit-cell vectors:

Diffraction limit #1: 2.669 (1.0000, 0.0000, 0.0000) 0.894 a* - 0.447 b*
Diffraction limit #2: 2.669 (0.0000, 1.0000, 0.0000) b*
Diffraction limit #3: 2.079 (0.0000, 0.0000, 1.0000) c*

Eigenvalues of overall anisotropy tensor on |F|s (Å^2) and corresponding eigenvectors of the overall anisotropy tensor as direction cosines in the orthogonal basis (standard PDB convention), and in terms of reciprocal unit-cell vectors:

Eigenvalue #1: 81.56 (1.0000, 0.0000, 0.0000) 0.894 a* - 0.447 b*
Eigenvalue #2: 81.56 (0.0000, 1.0000, 0.0000) b*
Eigenvalue #3: 28.46 (0.0000, 0.0000, 1.0000) c*

Statistics for the highest-resolution shell are shown in parentheses:

Wavelength (Å)	0.97857
Resolution range (Å)	25.45 - 2.219 (2.298 - 2.219)
Space group	P 6_5 2 2
Unit cell	29.387 Å 29.387 Å 295.649 Å 90° 90° 120°
Total reflections	8883 (825)
Unique reflections	4442 (46)
Multiplicity	2.0 (2.0)
Completeness (%)	66.67 (11.27)
Mean I/sigma(I)	9.38 (0.10)
Wilson B-factor (Å^2)	35.75
R_{merge}	0.01821 (2.691)
R_{meas}	0.02576 (3.805)
R_{pim}	0.01821 (2.691)
$CC_{1/2}$	1 (0.495)
CC	1 (0.814)
Reflections used in refinement	2965 (47)
Reflections used for R_{free}	298 (6)
R_{work}	0.3054 (0.4245)
R_{free}	0.3485 (0.3730)
$CC_{(work)}$	0.922 (0.512)
$CC_{(free)}$	0.856 (0.796)
Number of non-hydrogen atoms	517
macromolecules	512
ligands	0
solvent	5
Protein residues	62
RMS(bonds)	0.012
RMS(angles)	1.43
Ramachandran favored (%)	100.00
Ramachandran allowed (%)	0.00
Ramachandran outliers (%)	0.00
Rotamer outliers (%)	14.04
Clashscore	0.96
Average B-factor (Å^2)	52.96
macromolecules	53.11
solvent	37.63

Supplementary Table 2 – Crystallographic structure of the Myo6 dimerization domain – data and refinement statistics.

* Solvent content estimated by *Matthews_coef*[17]

Construct	primers	Cloning technique
	**for Sf9/baculovirus expression**	
FLMyo6 (WT)(NI)	5’CTGATTATCCTCAGCAAAACCCAGCAGCTCAGATTCCTGCC<br>5’GTTTTGCTGAGGATAATCAGTAACAGACTTTGGAGCACGTTG	PCR
FLMyo6 (WT)(SI)	5' CGACCTGGCGCTGCGGAGAGGTCCTGCTGTACTAGCCACC<br>5’ CCGCAGCGCCAGGTCGGCCTGGGCCTCATCACTG	Quikchange[18]
FLMyo6 (L926Q)	5’GAGGAAGCAGAAAGGCAGAGGCGTATTCAAGAAGAAATGGAAAAGG<br>5’GAATACGCCTCtGCCTTTCTGCTTCCTCTTCCTGCTG	Quikchange[18]
FLMyo6 (T888D.Q892E.V903D)	5' TTAAGTCCGATATGATGACGGAGGAACAAATCCAGAAAG<br>5' ATTTGTTCCTCCGTCATCATATCGGACTTAATTTTGGCCATC	Reverse PCR
FLMyo6 (SAHmimic)	Already published construct[19]	
MD (1-789)	Already published construct [20]	
MD^{Ins2} (2-816)	Already published construct [9]	
MD$^{Ins2/IQ/3HB}$(1-917)	Already published construct [21]	
MD$^{Ins2/IQ/3HB}$ T845C	Already published construct [22]	
MD$^{Ins2/IQ/3HB}$ T845C, A880C	Already published construct [22]	
zippered dimers A880C	Already published construct [22]	
zippered dimers T845C, A880C	Already published construct [22]	
zippered dimers T845C, A880C, L926Q	5’GAGGAAGCAGAAAGGCAGAGGCGTATTCAAGAAGAAATGGAAAAGG<br>5’GAATACGCCTCtGCCTTTCTGCTTCCTCTTCCTGCTG	Quikchange[18]
Jo-Myo6-In	Vector:<br>5'ATCGGGCGCGGATCAGATCTGCAGCGGCCGCCCACCATGTACCCCCAGACCGGCAC<br>5'GGAAAGGATCCCGGGTACCTTCTAGAATTCCACGTCCCTGATCTTGGGGATGG<br>Myo6 insert:<br>5' CGGCGGCAGCGGCATGGAGGATGGAAAGCCC<br>5' TTCCATCCTCCATGCCGCTGCCGCCGCTGCC<br>Jo-In insert:<br>5' GAGTCTGTTAAAGGGCGGCTCTGGCGGCTC<br>5' CGCCAGAGCCGCCCTTTAACAGACTCTGCAGC	Gibson[23]
	**for *Escherichia coli* expression**	
Ins2/IQ/3HB	Vector:<br>5'-TGACTCGAGGCATGCGGTAC-3'<br>5’-GGATCCCTGAAAATACAGGTTTTCG-3’<br>Insert:<br>5’-acctgtattttcagggatccAGAGTCAATCACTGGCTC-3’<br>5’-gtaccgcatgcctcgagtcaTTTTTTTTTCTGTAATGCACTG	Gibson[23]
^{YFP}CBD	5’ATGGCACTCCGAAGAGGTCCT<br>5’ACTTCCGCCCTGAAAATACAG	Reverse PCR
^{YFP}CBD (D1157V.Y1159D.D1161R.Q1163V)	5’TTCAGGACAAACGCCCTGTGAACAAGAAAAAGGGCTGGTGG<br>5’CCGCAGGGCGGATGAAGG	Reverse PCR
^{His}GIPC1	5’GATCTGCCGTCGGCCTTTG<br>5’TCCCATGGCGCCCTGAAAATAC	Reverse PCR
^{mNeonGreen}GIPC1	Vector:<br>5’ATGGCTGCCGCGCGGCAC<br>5’GGTAGTGGACATATGGGCCGAGGGAC<br>Insert:<br>5’ccgcgcggcagccatATGGTGAGCAAGGGCGAG<br>5’ccatatgtccactacccCTTGTACAGCTCGTCCATGC	Gibson[23]
^{His}TOM1 (207-end)	5’ GAAAACCTGTATTTTCAGGGC<br>5’GTGATGATGATGATGATGGC	Reverse PCR
TOM1 (436-461)	5’ GGGGTCACCAGCGAAGAATTTGAC<br>5’ GCCGCCTCCCATGGCGCTC	Reverse PCR
Dab2	a kind gift of Christopher Toseland	

Myo6 875-940	5’ TGACGGATCCCCTGAATTC<br>5’ ACGCCTCTTCCTTTCTTTC	PCR
Myo6 875-955	5’ CCCATATGGAAATTTCTATTGATGCTCTAATGGCC<br>5’ GGGGATCCGTCACCGCCTCTCCTCTTCCTCCTTC	PCR, digestion with NdeI and BamHI
Myo6 834-955	5’ GCCATATGAAACCACGGATTGACGGCCTGGTTAAG<br>5’ GTTTCGGATCCTACCGCCTCTCCTCTTCCTCC	PCR, digestion with NdeI and BamHI
Myo6 880-940	5’ GCTCTAATGGCCAAAATTAAG<br>5’ ACGCCTCTTCCTTTCTTTC	PCR
Myo6 888-940	5’ ACCATGATGACGAGGGAGCAGATACAGAAAG<br>5’ CATATGTATATCTCCTTCTTAAAGTTAAACAAAATTATTTC<br>5’ CACCACCACCACCACCACTGAGATCC<br>5’ ACGCCTCTTCCTTTCTTTCTCCATCTCC	2 step PCR
Myo6 912-end	5’GCACTTGTTAAAAGCTCAGAAGATCTCCTCAG<br>5’GGAATGGTGATGGTGGTGGTGCATGG	Reverse PCR
Myo6 rTEV-875-940	Vector:<br>5’GGATCCCTGAAAATACAGGTTTTCGG<br>5’CTCGAGGCATGCGGTACC<br>Insert:<br>5’acctgtattttcagggatccATGGAAATTTCTATTGATGCTC<br>5’ttggtaccgcatgcctcgagTCAACGCCTCTTCCTTTC	Gibson[23]
Myo6 rTEV-875-940<br>T888D R892E V903D	5’AAGTCCGACATGATGACGGAGGAGCAGATACAGAAAGAGTATGATGC<br>5’MACTTGATAAAAGCTCAGAAGATCTCCTCAGTGCACTGC	Quikchange[18]
	**for studying Myo6 recruitment at melanosomes**	
FLMyo6 (SAHmimic)	5’TTCTTCTTCTTCACGACGTTTACGTTCTTCCTCTTCCTGCTGTTTTTTTTTC<br>5’CGTAAAAAACGTGAGGAAGAAGAAGAAAGAAAAAGACGTGAAGAAGAC<br>G	Reverse PCR
FLMyo6 (L926Q)	5’GAGGAAGCAGAAAGGCAGAGGCGTATTCAAGAAGAAATGGAAAAGG<br>5’GAATACGCCTCtGCCTTTCTGCTTCCTCTTCCTGCTG	Quikchange[18]
FLMyo6 (SAHmimic.I1072A)	5’ATCACAAGAAGTATTGATGGTATCACG<br>5’GCCGAGCTCCTGGCAGCTTGC	Reverse PCR
FLMyo6 (L926Q.I1072A)	5’ATCACAAGAAGTATTGATGGTATCACG<br>5’GCCGAGCTCCTGGCAGCTTGC	Reverse PCR
FLMyo6 (I1072A)	5’ATCACAAGAAGTATTGATGGTATCACG<br>5’GCCGAGCTCCTGGCAGCTTGC	Reverse PCR
Jo-Myo6-In (I1072A)	5’ATCACAAGAAGTATTGATGGTATCACG<br>5’GCCGAGCTCCTGGCAGCTTGC	Reverse PCR
CBD (I1072A)	5’ATCACAAGAAGTATTGATGGTATCACG<br>5’GCCGAGCTCCTGGCAGCTTGC	Reverse PCR
MST-TOM1 (299-end)	5’GAACGGTTTGAACGGTTC<br>5’TTTGGAAGTGAGTGAATTCG	Reverse PCR
MST-Dab2 (650-end)	Vector:<br>5’AAGTTGCACTAACCTTGCAG<br>5’TTTGGAAGTGAGTGAATTCG<br>Insert:<br>5’AATTCACTCACTTCCAAAGATGTGAAAGAGATGTTCAAAGATTTTC<br>5’GCAAGGTTAGTGCAACTTTTAGGCAAACGGATTACCAAAC	Gibson[23]
	**for the transferrin uptake assays**	
FLMyo6 (WT)	Vector:<br>5’GGACGAGCTGTACAAGTAGGGTACCGAGCTCGGATCCAC<br>5’CAAACGGGTTTTCCATCCTCCATggtggcAAGCTTAAGTTTAAAC<br>Insert1 (Myo6):	Gibson[23]

	5’GTTTAAACTTAAGCTTgccaccATGGAGGATGGAAAACCCGTTTG<br>5’CTTGCTCACGCCtcggccgcccttgagcaggttctgcagc<br>Insert2 (mApple):<br>5’gctgcagaacctgctcaagggcggccgaGGCGTGAGCAAG<br>5’GTGGATCCGAGCTCGGTACCCTACTTGTACAGCTCGTCC	
FLMyo6 (T888D.R892E.V903E)	For T888D:<br>5’CcaaaattaagtccGAcatgatgacgaggg<br>5’ccctcgtcatcatgTCggacttaattttgg<br>For R892D:<br>5’CcGAcatgatgacgGAggagcagatacag<br>5’ctgtatctgctccTCcgtcatcatgTCgg<br>For V903D:<br>5’GtatgatgcacttgACaaaagctcagaag<br>5’cttctgagcttttGTcaagtgcatcatac	3 successive PCR

Supplementary Table 3 – Primers and cloning techniques used to clone all the constructs required for this study.

Construct	IMAC	SEC
Ins2/IQ/3HB*	20 mM Tris pH 7.5; 150 mM NaCl; 1 mM DTT (+ 4 and 300 mM Imidazole for binding/washing and elution, respectively)	20 mM HEPES, pH 7.5; 50 mM NaCl; 2.5mM $MgCl_2$; 1 mM TCEP
^{YFP}CBD (WT and mutant)	20 mM Tris pH 7.5; 150 mM KCl; 40 mM Imidazole pH 7.5; 5% Glycerol; 1mM DTT (+300 mM NaCl in the washing step; and 100 mM KCl; 300 mM imidazole; 1 mM TCEP for elution)	20 mM Tris pH 7.5; 100 mM KCl; 1 mM TCEP
GIPC1	20 mM HEPES pH 7.5; 150 mM NaCl; 1 mM DTT (+4 or 200 mM Imidazole for binding/washing and elution, respectively)	50 mM HEPES pH 7.5; 150 mM or 50 mM NaCl; 1 mM DTT
TOM1	20 mM Tris pH 7.5; 300 mM NaCl; 1 mM DTT (+4 or 300 mM Imidazole for binding/washing and elution, respectively)	20 mM HEPES pH 7.5; 150 mM NaCl; 1 mM DTT
Dab2	20 mM Tris pH 8.5; 300 mM NaCl; 1 mM DTT (+4 or 200 mM Imidazole for binding/washing and elution, respectively)	20 mM Tris pH 8.5; 150 mM NaCl; 1 mM DTT
Myo6 875-940, 875-955**, 880-940, 888-940** and 834-955	20 mM Tris, pH7.5; 150 mM NaCl (+4 or 200 mM Imidazole for binding/washing and elution, respectively)	10 mM Tris, pH7.5 ; 50mM NaCl
Myo6 875-940 triple mutant**	20 mM Tris, pH7.5; 600 mM NaCl (+4 or 200 mM Imidazole for binding/washing and elution, respectively)	10 mM Tris, pH7.5 ; 300mM NaCl

Supplementary Table 4 – Purification buffers used for constructs expressed in *E. Coli*

* presence of 2 CaM bound was confirmed by SEC-MALS (not shown).
** Ion exchange in MonoQ 5/50 GL prior to SEC.
*** Ion exchange in MonoS 5/50 GL prior to SEC.
(For constructs including a rTEV cleavage site, homemade rTEV was added 1:50 m/m, and a second passage through IMAC was performed before SEC)

Supplementary References

1. Manalastas-Cantos, K. *et al.* ATSAS 3.0: expanded functionality and new tools for small-angle scattering data analysis. *J Appl Cryst* **54**, 343–355 (2021).
2. Svergun, D. I., Petoukhov, M. V. & Koch, M. H. Determination of domain structure of proteins from X-ray solution scattering. *Biophys J* **80**, 2946–2953 (2001).
3. Volkov, V. V., Svergun, D. I., & IUCr. Uniqueness of ab initio shape determination in small-angle scattering. *Journal of Applied Crystallography* vol. 36 860–864 http://scripts.iucr.org/cgi-bin/paper?S0021889803000268 (2003).
4. Petoukhov, M. V. *et al.* New developments in the ATSAS program package for small-angle scattering data analysis. *J Appl Crystallogr* **45**, 342–350 (2012).
5. Schneidman-Duhovny, D., Hammel, M., Tainer, J. A. & Sali, A. FoXS, FoXSDock and MultiFoXS: Single-state and multi-state structural modeling of proteins and their complexes based on SAXS profiles. *Nucleic Acids Res* **44**, W424-429 (2016).
6. Schneidman-Duhovny, D., Hammel, M., Tainer, J. A. & Sali, A. Accurate SAXS profile computation and its assessment by contrast variation experiments. *Biophys J* **105**, 962–974 (2013).
7. Franke, D. *et al.* ATSAS 2.8: a comprehensive data analysis suite for small-angle scattering from macromolecular solutions. *J Appl Crystallogr* **50**, 1212–1225 (2017).
8. Berggren, K. *et al.* Background-free, high sensitivity staining of proteins in one- and two-dimensional sodium dodecyl sulfate-polyacrylamide gels using a luminescent ruthenium complex. *Electrophoresis* **21**, 2509–2521 (2000).
9. Ménétrey, J. *et al.* The structure of the myosin VI motor reveals the mechanism of directionality reversal. *Nature* **435**, 779–785 (2005).
10. Bonnet, J. *et al.* Autocatalytic association of proteins by covalent bond formation: a Bio Molecular Welding toolbox derived from a bacterial adhesin. *Sci Rep* **7**, 43564 (2017).
11. Panjkovich, A. & Svergun, D. I. Deciphering conformational transitions of proteins by small angle X-ray scattering and normal mode analysis. *Phys Chem Chem Phys* **18**, 5707–5719 (2016).
12. Wienken, C. J., Baaske, P., Rothbauer, U., Braun, D. & Duhr, S. Protein-binding assays in biological liquids using microscale thermophoresis. *Nat Commun* **1**, 100 (2010).
13. Barnes, C. A. *et al.* Remarkable Rigidity of the Single α-Helical Domain of Myosin-VI As Revealed by NMR Spectroscopy. *J Am Chem Soc* **141**, 9004–9017 (2019).
14. Kim, H., Hsin, J., Liu, Y., Selvin, P. R. & Schulten, K. Formation of salt bridges mediates internal dimerization of myosin VI medial tail domain. *Structure* **18**, 1443–1449 (2010).
15. Emsley, P. & Cowtan, K. Coot: model-building tools for molecular graphics. *Acta Crystallogr D Biol Crystallogr* **60**, 2126–2132 (2004).
16. Varadi, M. *et al.* AlphaFold Protein Structure Database: massively expanding the structural coverage of protein-sequence space with high-accuracy models. *Nucleic Acids Res* **50**, D439–D444 (2022).
17. Matthews, B. W. Solvent content of protein crystals. *J Mol Biol* **33**, 491–497 (1968).
18. Liu, H. & Naismith, J. H. An efficient one-step site-directed deletion, insertion, single and multiple-site plasmid mutagenesis protocol. *BMC Biotechnol* **8**, 91 (2008).
19. Mukherjea, M. *et al.* Myosin VI must dimerize and deploy its unusual lever arm in order to perform its cellular roles. *Cell Rep* **8**, 1522–1532 (2014).
20. Ménétrey, J., Llinas, P., Mukherjea, M., Sweeney, H. L. & Houdusse, A. The structural basis for the large powerstroke of myosin VI. *Cell* **131**, 300–308 (2007).
21. Mukherjea, M. *et al.* Myosin VI dimerization triggers an unfolding of a three-helix bundle in order to extend its reach. *Mol Cell* **35**, 305–315 (2009).
22. De La Cruz, E. M., Ostap, E. M. & Sweeney, H. L. Kinetic mechanism and regulation of myosin VI. *J Biol Chem* **276**, 32373–32381 (2001).
23. Gibson, D. G. *et al.* Enzymatic assembly of DNA molecules up to several hundred kilobases. *Nat Methods* **6**, 343–345 (2009).
