# Supplementary material for: How myosin VI traps its off-state, is activated and dimerizes
Source: Nat Commun. 2023 Oct 23;14:6732. doi: 10.1038/s41467-023-42376-2 (PMC10593786; doi:10.1038/s41467-023-42376-2)
Supplement: Supplementary file 3 — Description of Additional Supplementary Files [file 41467_2023_42376_MOESM3_ESM.pdf]

Description of Additional Supplementary Files

Supplementary Movie 1: 3D view of the Myo6 off-state docked in the negative staining reconstruction (from Fig. 3A).

Supplementary Movie 2: Representation of the crystal packing of the dimerization Myo6 fragment 875-937 as calculated with PyMol. Each crystallographic dimer is represented in a different color.
