# Supplementary material for: How myosin VI traps its off-state, is activated and dimerizes
Source: Nat Commun. 2023 Oct 23;14:6732. doi: 10.1038/s41467-023-42376-2 (PMC10593786; doi:10.1038/s41467-023-42376-2)
Supplement: Supplementary file 6 — Reporting Summary [file 41467_2023_42376_MOESM6_ESM.pdf]

nature portfolio

Corresponding author(s): Anne Houdusse and H. Lee Sweeney

Last updated by author(s): Oct 5, 2023

Reporting Summary

Nature Portfolio wishes to improve the reproducibility of the work that we publish. This form provides structure for consistency and transparency in reporting. For further information on Nature Portfolio policies, see our Editorial Policies and the Editorial Policy Checklist.

Statistics

For all statistical analyses, confirm that the following items are present in the figure legend, table legend, main text, or Methods section.

n/a	Confirmed	
☐	☒	The exact sample size (*n*) for each experimental group/condition, given as a discrete number and unit of measurement
☐	☒	A statement on whether measurements were taken from distinct samples or whether the same sample was measured repeatedly
☐	☒	The statistical test(s) used AND whether they are one- or two-sided<br>*Only common tests should be described solely by name; describe more complex techniques in the Methods section.*
☒	☐	A description of all covariates tested
☐	☒	A description of any assumptions or corrections, such as tests of normality and adjustment for multiple comparisons
☐	☒	A full description of the statistical parameters including central tendency (e.g. means) or other basic estimates (e.g. regression coefficient) AND variation (e.g. standard deviation) or associated estimates of uncertainty (e.g. confidence intervals)
☐	☒	For null hypothesis testing, the test statistic (e.g. *F*, *t*, *r*) with confidence intervals, effect sizes, degrees of freedom and *P* value noted<br>*Give P values as exact values whenever suitable.*
☒	☐	For Bayesian analysis, information on the choice of priors and Markov chain Monte Carlo settings
☒	☐	For hierarchical and complex designs, identification of the appropriate level for tests and full reporting of outcomes
☒	☐	Estimates of effect sizes (e.g. Cohen's *d*, Pearson's *r*), indicating how they were calculated

Our web collection on statistics for biologists contains articles on many of the points above.

Software and code

Policy information about availability of computer code

Data collection	For Myo6 localisation on melanosomes assays: Metamorph 7.10.1 software; Molecular Devices . For transferrin uptake assays: Leica Application Suite X v3.5.23225.7
Data analysis	For cell-based assays: FiJi (v2.1.0/1.53n) for IF images analysis and code generation. GraphPad Prism 9.4.1 for statistical analysis. For SAXS assays: Foxtrot 3.5.10-3979, ATSAS 3.0, ATSAS online and FoXS server were used for data processing and analysis. For SEC-MALS assays: Astra 6.1.7 (Wyatt Technologies) was used for data analysis. For MST assays: NTAffinity Analysis v2.0.2 was used for data anlysis (NanoTemper). For pull-down assays: ImageLab 6.1.0 build 7 Standard Edition (Bio-Rad Laboratories, Inc.) was used for data analysis. For X-ray crystallization: XDS 20180409, autoPROC 1.0.5 (20180515), Staraniso 1.10.15 and Buster 2.10.4 (8-Jun-2022) were used for data processing. For negative staining assays: CryoSPARC v2.12 (nov/2019) was used for data analysis. For crosslink-mass spectroscopy experiment: Xcalibur 4.1.31.9, ProteoWizard 3.0.22215 and MeroX 2.0 were used for data analysis.

For manuscripts utilizing custom algorithms or software that are central to the research but not yet described in published literature, software must be made available to editors and reviewers. We strongly encourage code deposition in a community repository (e.g. GitHub). See the Nature Portfolio guidelines for submitting code & software for further information.

Data

Policy information about availability of data

All manuscripts must include a data availability statement. This statement should provide the following information, where applicable:

- Accession codes, unique identifiers, or web links for publicly available datasets
- A description of any restrictions on data availability
- For clinical datasets or third party data, please ensure that the statement adheres to our policy

The atomic model of the Myo6 proximal dimer generated in this study is available on the PDB66 under the accession code 8ARD [https://doi.org/10.2210/pdb8ard/pdb]. The other atomic coordinates used in this study are available on the PDB66 under the accession numbers 3H8D [https://doi.org/10.2210/pdb3h8d/pdb], 6J56 [https://doi.org/10.2210/pdb6J56/pdb], 5V6E [https://doi.org/10.2210/pdb5V6E/pdb], 2LD3 [https://doi.org/10.2210/pdb2ld3/pdb], 4ANJ [https://doi.org/10.2210/pdb4ANJ/pdb], 3GN4 [https://doi.org/10.2210/pdb3GN4/pdb], 5MKC [https://doi.org/10.2210/pdb5MKC/pdb], 6OBI [https://doi.org/10.2210/pdb6OBI/pdb] and 6BNP [https://doi.org/10.2210/pdb6BNP/pdb]. The mass spectroscopy data supporting Sup Fig. 7 data have been deposited to the ProteomeXchange Consortium via the PRIDE67 partner repository with the dataset identifier PXD044767. Source data are provided with this paper.

Human research participants

Policy information about studies involving human research participants and Sex and Gender in Research.

Reporting on sex and gender	n/a
Population characteristics	n/a
Recruitment	n/a
Ethics oversight	n/a

Note that full information on the approval of the study protocol must also be provided in the manuscript.

Field-specific reporting

Please select the one below that is the best fit for your research. If you are not sure, read the appropriate sections before making your selection.

☒ Life sciences ☐ Behavioural & social sciences ☐ Ecological, evolutionary & environmental sciences

For a reference copy of the document with all sections, see nature.com/documents/nr-reporting-summary-flat.pdf

Life sciences study design

All studies must disclose on these points even when the disclosure is negative.

Sample size

Sample size is shown in figure legends. For transferrin uptake assay, no sample size was pre-determined. The number of cells analyzed vary from 15-43 per replicate per condition for a total of 58-79 cells analyzed per condition. These values are comparable to fluorescence-based transferrin uptake assay reported a recent Nat Commun report:
Rai, A., Singh, A.K., Bleimling, N. et al. Rep15 interacts with several Rab GTPases and has a distinct fold for a Rab effector. Nat Commun 13, 4262 (2022). https://doi.org/10.1038/s41467-022-31831-1
For the characterization of Myo6 recruitment at melanosomes, ~20-30 cells were analyzed per conditions for each replicate. These values are comparable to the values of a previous study on MNT-1 cells:
Ripoll L, Heiligenstein X, Hurbain I, Domingues L, Figon F, Petersen KJ, Dennis MK, Houdusse A, Marks MS, Raposo G, Delevoye C. Myosin VI and branched actin filaments mediate membrane constriction and fission of melanosomal tubule carriers. J Cell Biol. 2018 Aug 6;217(8):2709-2726. doi: 10.1083/jcb.201709055. Epub 2018 Jun 6. PMID: 29875258; PMCID: PMC6080934.
In both cases, statistical relevance was checked by calculating p value.

Data exclusions

For cell-based assays on MNT-1: Non or less-pigmented cells, cells with low fluorescence intensity and/or low expression precluding signal thresholding and further quantification were excluded) (~11% of cells excluded)

Replication

All replicates are reported in the manuscript. One replicate was excluded from this study for ATPase and MST assays due to protein degradation (identified on SDS-page after the experiment). Other replicates for all our experiments were succesfull and thus included in the manuscript.

Randomization

For the characterization of Myo6 recruitment at melanosomes, only cells for which both Myo6 construct and MST construct transfection were successfull were taken into account.
All fluorescence images for the transferrin uptake assay were acquired from random fields of view.
For Myo6 dimer structure determination, 10% data were randomly selected and used to measure the model-to-data agreement.

Blinding	Investigators were not blinded due to the unbiased nature of the software based analysis performed.

Reporting for specific materials, systems and methods

We require information from authors about some types of materials, experimental systems and methods used in many studies. Here, indicate whether each material, system or method listed is relevant to your study. If you are not sure if a list item applies to your research, read the appropriate section before selecting a response.

Materials & experimental systems

n/a	Involved in the study
☐	☒ Antibodies
☐	☒ Eukaryotic cell lines
☒	☐ Palaeontology and archaeology
☒	☐ Animals and other organisms
☒	☐ Clinical data
☒	☐ Dual use research of concern

Methods

n/a	Involved in the study
☒	☐ ChIP-seq
☒	☐ Flow cytometry
☒	☐ MRI-based neuroimaging

Antibodies

Antibodies used	Anti-MyoVI (rb pc)(EMD-Millipore, Cat# ABT42, Lot# 3011368, used at 1:1000 dilution for WB) and anti-DS-Red (rb pc) (TakaraBio, Cat# 632496, Lot# 2103116, used at 1:4000 dilution for IF).
Validation	Antibodies validation:<br>- Takara Bio anti-dsRed polyclonal antibody:<br>o previously validated for western blots against various red fluorescent protein including mOrange from which mApple was derived (provided by manufacturer)<br>- EMD Millipore anti-MyosinVI polyclonal antibody:<br>o previously validated for detection of myosin VI by western blots and immunofluorescence in A431 and NIH/3T3 cells (provided by manufacturer)<br>o western blots of lysates generated from control and myosin VI KO HeLa cells (Fig. S17A) provide further validation of the antibody's specificity

Eukaryotic cell lines

Policy information about cell lines and Sex and Gender in Research

Cell line source(s)	MNT-1 cells (human pigmented melanoma cell line derived from a male patient): a gift from Pr. Michael S. Marks (Department of Pathology and Laboratory Medicine, Children's Hospital of Philadelphia Research Institute, Philadelphia, PA; Department of Pathology and Laboratory Medicine and Department of Physiology, Perelman School of Medicine, University of Pennsylvania, Philadelphia, PA). HeLa (epithelial cells derived from a cervical carcinoma of a female patient): American Type Culture Collection (ATCC, Cat# CCL-2). Myo6 null HeLa: in-house generation from Hela cells purchased directly from ATCC (Cat# CCL-2).
Authentication	The MNT-1 cell line used in the manuscript was not authenticated. Inactivation of hMyo6 gene in HeLa cells was confirmed by PCR of DNA isolated from myo6 null HeLa cells using primers flanking the targeted genomic sequences. Loss of Myo6 expression was confirmed by western blot.
Mycoplasma contamination	The cell lines used in this study were regularly tested negative for mycoplasma contamination.
Commonly misidentified lines (See ICLAC register)	The cell lines used in the manuscript are not listed in the database of commonly misidentified cell lines maintained by ICLAC.

Palaeontology and Archaeology

Specimen provenance	*Provide provenance information for specimens and describe permits that were obtained for the work (including the name of the issuing authority, the date of issue, and any identifying information). Permits should encompass collection and, where applicable, export.*
Specimen deposition	*Indicate where the specimens have been deposited to permit free access by other researchers.*

Dating methods | *If new dates are provided, describe how they were obtained (e.g. collection, storage, sample pretreatment and measurement), where they were obtained (i.e. lab name), the calibration program and the protocol for quality assurance OR state that no new dates are provided.*

☐ Tick this box to confirm that the raw and calibrated dates are available in the paper or in Supplementary Information.

Ethics oversight | *Identify the organization(s) that approved or provided guidance on the study protocol, OR state that no ethical approval or guidance was required and explain why not.*

Note that full information on the approval of the study protocol must also be provided in the manuscript.

Animals and other research organisms

Policy information about studies involving animals; ARRIVE guidelines recommended for reporting animal research, and Sex and Gender in Research

Laboratory animals | *For laboratory animals, report species, strain and age OR state that the study did not involve laboratory animals.*

Wild animals | *Provide details on animals observed in or captured in the field; report species and age where possible. Describe how animals were caught and transported and what happened to captive animals after the study (if killed, explain why and describe method; if released, say where and when) OR state that the study did not involve wild animals.*

Reporting on sex | *Indicate if findings apply to only one sex; describe whether sex was considered in study design, methods used for assigning sex. Provide data disaggregated for sex where this information has been collected in the source data as appropriate; provide overall numbers in this Reporting Summary. Please state if this information has not been collected. Report sex-based analyses where performed, justify reasons for lack of sex-based analysis.*

Field-collected samples | *For laboratory work with field-collected samples, describe all relevant parameters such as housing, maintenance, temperature, photoperiod and end-of-experiment protocol OR state that the study did not involve samples collected from the field.*

Ethics oversight | *Identify the organization(s) that approved or provided guidance on the study protocol, OR state that no ethical approval or guidance was required and explain why not.*

Note that full information on the approval of the study protocol must also be provided in the manuscript.

Clinical data

Policy information about clinical studies

All manuscripts should comply with the ICMJE guidelines for publication of clinical research and a completed CONSORT checklist must be included with all submissions.

Clinical trial registration | *Provide the trial registration number from ClinicalTrials.gov or an equivalent agency.*

Study protocol | *Note where the full trial protocol can be accessed OR if not available, explain why.*

Data collection | *Describe the settings and locales of data collection, noting the time periods of recruitment and data collection.*

Outcomes | *Describe how you pre-defined primary and secondary outcome measures and how you assessed these measures.*

Dual use research of concern

Policy information about dual use research of concern

Hazards

Could the accidental, deliberate or reckless misuse of agents or technologies generated in the work, or the application of information presented in the manuscript, pose a threat to:

No	Yes	
☐	☐	Public health
☐	☐	National security
☐	☐	Crops and/or livestock
☐	☐	Ecosystems
☐	☐	Any other significant area

Experiments of concern

Does the work involve any of these experiments of concern:

No	Yes	
☐	☐	Demonstrate how to render a vaccine ineffective
☐	☐	Confer resistance to therapeutically useful antibiotics or antiviral agents
☐	☐	Enhance the virulence of a pathogen or render a nonpathogen virulent
☐	☐	Increase transmissibility of a pathogen
☐	☐	Alter the host range of a pathogen
☐	☐	Enable evasion of diagnostic/detection modalities
☐	☐	Enable the weaponization of a biological agent or toxin
☐	☐	Any other potentially harmful combination of experiments and agents

ChIP-seq

Data deposition

☐ Confirm that both raw and final processed data have been deposited in a public database such as GEO.

☐ Confirm that you have deposited or provided access to graph files (e.g. BED files) for the called peaks.

Data access links<br>*May remain private before publication.*	*For "Initial submission" or "Revised version" documents, provide reviewer access links. For your "Final submission" document, provide a link to the deposited data.*
Files in database submission	*Provide a list of all files available in the database submission.*
Genome browser session<br>(e.g. UCSC)	*Provide a link to an anonymized genome browser session for "Initial submission" and "Revised version" documents only, to enable peer review. Write "no longer applicable" for "Final submission" documents.*

Methodology

Replicates	*Describe the experimental replicates, specifying number, type and replicate agreement.*
Sequencing depth	*Describe the sequencing depth for each experiment, providing the total number of reads, uniquely mapped reads, length of reads and whether they were paired- or single-end.*
Antibodies	*Describe the antibodies used for the ChIP-seq experiments; as applicable, provide supplier name, catalog number, clone name, and lot number.*
Peak calling parameters	*Specify the command line program and parameters used for read mapping and peak calling, including the ChIP, control and index files used.*
Data quality	*Describe the methods used to ensure data quality in full detail, including how many peaks are at FDR 5% and above 5-fold enrichment.*
Software	*Describe the software used to collect and analyze the ChIP-seq data. For custom code that has been deposited into a community repository, provide accession details.*

Flow Cytometry

Plots

Confirm that:

☐ The axis labels state the marker and fluorochrome used (e.g. CD4-FITC).

☐ The axis scales are clearly visible. Include numbers along axes only for bottom left plot of group (a 'group' is an analysis of identical markers).

☐ All plots are contour plots with outliers or pseudocolor plots.

☐ A numerical value for number of cells or percentage (with statistics) is provided.

Methodology

Sample preparation	*Describe the sample preparation, detailing the biological source of the cells and any tissue processing steps used.*
Instrument	*Identify the instrument used for data collection, specifying make and model number.*

Software	*Describe the software used to collect and analyze the flow cytometry data. For custom code that has been deposited into a community repository, provide accession details.*
Cell population abundance	*Describe the abundance of the relevant cell populations within post-sort fractions, providing details on the purity of the samples and how it was determined.*
Gating strategy	*Describe the gating strategy used for all relevant experiments, specifying the preliminary FSC/SSC gates of the starting cell population, indicating where boundaries between "positive" and "negative" staining cell populations are defined.*

☐ Tick this box to confirm that a figure exemplifying the gating strategy is provided in the Supplementary Information.

Magnetic resonance imaging

Experimental design

Design type	*Indicate task or resting state; event-related or block design.*
Design specifications	*Specify the number of blocks, trials or experimental units per session and/or subject, and specify the length of each trial or block (if trials are blocked) and interval between trials.*
Behavioral performance measures	*State number and/or type of variables recorded (e.g. correct button press, response time) and what statistics were used to establish that the subjects were performing the task as expected (e.g. mean, range, and/or standard deviation across subjects).*

Acquisition

Imaging type(s)	*Specify: functional, structural, diffusion, perfusion.*
Field strength	*Specify in Tesla*
Sequence & imaging parameters	*Specify the pulse sequence type (gradient echo, spin echo, etc.), imaging type (EPI, spiral, etc.), field of view, matrix size, slice thickness, orientation and TE/TR/flip angle.*
Area of acquisition	*State whether a whole brain scan was used OR define the area of acquisition, describing how the region was determined.*

Diffusion MRI ☐ Used ☐ Not used

Preprocessing

Preprocessing software	*Provide detail on software version and revision number and on specific parameters (model/functions, brain extraction, segmentation, smoothing kernel size, etc.).*
Normalization	*If data were normalized/standardized, describe the approach(es): specify linear or non-linear and define image types used for transformation OR indicate that data were not normalized and explain rationale for lack of normalization.*
Normalization template	*Describe the template used for normalization/transformation, specifying subject space or group standardized space (e.g. original Talairach, MNI305, ICBM152) OR indicate that the data were not normalized.*
Noise and artifact removal	*Describe your procedure(s) for artifact and structured noise removal, specifying motion parameters, tissue signals and physiological signals (heart rate, respiration).*
Volume censoring	*Define your software and/or method and criteria for volume censoring, and state the extent of such censoring.*

Statistical modeling & inference

Model type and settings	*Specify type (mass univariate, multivariate, RSA, predictive, etc.) and describe essential details of the model at the first and second levels (e.g. fixed, random or mixed effects; drift or auto-correlation).*
Effect(s) tested	*Define precise effect in terms of the task or stimulus conditions instead of psychological concepts and indicate whether ANOVA or factorial designs were used.*

Specify type of analysis: ☐ Whole brain ☐ ROI-based ☐ Both

Statistic type for inference (See Eklund et al. 2016)	*Specify voxel-wise or cluster-wise and report all relevant parameters for cluster-wise methods.*
Correction	*Describe the type of correction and how it is obtained for multiple comparisons (e.g. FWE, FDR, permutation or Monte Carlo).*

Models & analysis

n/a	Involved in the study
☐	☐ Functional and/or effective connectivity
☐	☐ Graph analysis
☐	☐ Multivariate modeling or predictive analysis

Functional and/or effective connectivity	*Report the measures of dependence used and the model details (e.g. Pearson correlation, partial correlation, mutual information).*
Graph analysis	*Report the dependent variable and connectivity measure, specifying weighted graph or binarized graph, subject- or group-level, and the global and/or node summaries used (e.g. clustering coefficient, efficiency, etc.).*
Multivariate modeling and predictive analysis	*Specify independent variables, features extraction and dimension reduction, model, training and evaluation metrics.*
