# Supplementary material for: How myosin VI traps its off-state, is activated and dimerizes
Source: Nat Commun. 2023 Oct 23;14:6732. doi: 10.1038/s41467-023-42376-2 (PMC10593786; doi:10.1038/s41467-023-42376-2)
Supplement: Supplementary file 7 — Source Data [file 41467_2023_42376_MOESM7_ESM.zip › source data files/SupFig4B MS slower band.pdf]

Protein Entry: Q9UM54-8
Peptide matches for MS/MS Ions Search (MS/MS) Analysis C8876VM.

Name in myProMS : **Q9UM54-8**
Original identifier : Q9UM54-8
Description : no description
Nominal mass (Mr) : 175567 Da (1532 aa)
Species : *unknown organism*
Last modified : Never Edit Protein

Show **Synonyms or isoforms of Q9UM54-8 in Project**

Show **List of Analyses where Q9UM54-8 is found**

Show **List of Quantifications where Q9UM54-8 is found**

Show **Check if Q9UM54-8 shares peptides with other proteins in Project**

Show **Post-translational modifications relevant to Project**

Hide **Detailed sequence coverage in Analysis C8876VM**

(Matching peptides are shown in **bold red**, overlapping peptides in **bold blue**). Extract sequence
Extract covered sequence

```
  &                                               *
1 MYPQTGTYPD VQTPYQIIKV DGSEKNGQHK ALNPNPYERV IPEGTLSKRI YQVNNLDDNQ
61 YGIELTVSGK TVYEGGSGGS GGSGMEDGKP VWAPHPTDGF QMGNIVDIGP DSLTIEPLNQ
                       *     *  *
121 KGKTFLALIN QVFPAEEDSK KDVEDNCSLM YLNEATLLHN IKVRYSKDRI YTYVANILIA
                         *                    &
181 VNPYFDIPKI YSSEAIKSYQ GKSLGTRPPH VFAIADKAFR DMKVLKMSQS IIVSGESGAG
              *
241 KTENTKFVLR YLTESYGTGQ DIDDRIVEAN PLLEAFGNAK TVRNNNSSRF GKFVEIHFNE
     *                                  *
301 KSSVVGGFVS HYLLEKSRIC VQGKEERNYH IFYRLCAGAS EDIREKLHLS SPDNFRYLNR
                                *  *
361 GCTRYFANKE TDKQILQNRK SPEYLKAGSM KDPLLDDHGD FIRMCTAMKK IGLDDEEKLD
       *                    *          *                         *
421 LFRVVAGVLH LGNIDFEEAG STSGGCNLKN KSAQSLEYCA ELLGLDQDDL RVSLTTRVML
                                   *
481 TTAGGTKGTV IKVPLKVEQA NNARDALAKT VYSHLFDHVV NRVNQCFPFE TSSYFIGVLD
```

541 IAGFEYFEHN SFEQFCINYC NEKLQQFFNE RILKEEQELY QKEGLGVNEV HYVDNQDCID

601 LIEAKLVGIL DILDEENRLP QPSDQHFTSA VHQKHKDHFR LTIPRKSKLA VHRNIRDDEG

661 FIIRHFAGAV CYETTQFVEK NNDALHMSLE SLICESRDKF IRELFESSTN NNKDTKQKAG

721 KLSFISVGNK FKTQLNLLLD KLRSTGASFI RCIKPNLKMT SHHFEGAQIL SQLQCSGMVS

781 VLDLMQGGYP SRASFHELYN MYKKYMPDKL ARLDPRLFCK ALFKALGLNE NDYKFGLTKV

841 FFRPGKFAEF DQIMKSDPDH LAELVKRVNH WLTCSRWKKV QWCSLSVIKL KNKIKYRAEA

901 CIKMQKTIRM WLCKRRHKPR IDGLVKVGTL KKRLDKFNEV VSVLKDGKPE MNKQIKNLEI

961 SIDTLMAKIK STMMTQEQIQ KEYDALVKSS EELLSALQKK KQQEEEAERL RRIQEEMEKE

1021 RKRREEDEKR RRKEEEERRM KLEMEAKRKQ EEEERKKRED DEKRIQAEVE AQLARQKEEE

1081 SQQQAVLEQE RRDRELALRI AQSEAELISD EAQADLALRR SLDSYPVSKN DGTRPKMTPE

1141 QMAKEMSEFL SRGPAVLATK AAAGTKKYDL SKWKYAELRD TINTSCDIEL LAACREEFHR

1201 RLKVYHAWKS KNKKRNTETE QRAPKSVTDY DFAPFLNNSP QQNPAAQIPA RQREIEMNRQ

1261 QRFFRIPFIR PADQYKDPQS KKKGWWYAHF DGPWIARQME LHPDKPPILL VAGKDDMEMC

1321 ELNLEETGLT RKRGAEILPR QFEEIWERCG GIQYLQNAIE SRQARPTYAT AMLQSLLKGG

1381 SGGSGGSGTE KKSIENGTIT DPMGELIDLQ LGTDGRFDPA DYTLTANDGS RLENGQAVGG

1441 PQNDGGLLKN AKVLYDTTEK RIRVTGLYLG TDEKVTLTYN VRLNDEFVSN KFYDTNGRTT

1501 LHPKEVEQNT VRDFPIPKIR DVELDYKDDD DK

Protein score: 11956.3
Best peptide specificity: 100 %
Peptide coverage: 68.4 %

Show **Peptide list** ⊞

Show **Proteins interacting with Q9UM54-8 in selected Analyses**

Show **General features of Q9UM54-8**

Show **Links to external resources for Q9UM54-8**
